# Extracellular Vesicles in Autoimmune Diseases: From Diagnostic Biomarkers to Engineered Therapeutics

**DOI:** 10.1002/advs.202521802

**Published:** 2026-02-12

**Authors:** Yufei Wu, Huiyuan Ma, Tinghui Zhu, Shuwei Huang, Manxi Lu, Qiong Wang, Zai‐Long Chi

**Affiliations:** ^1^ Eye Hospital of Wenzhou Medical University Wenzhou Zhejiang China

**Keywords:** autoimmune diseases, extracellular vesicles, isolation and engineering, mechanism of action, therapeutic applications

## Abstract

Autoimmune diseases (ADs) are chronic disorders caused by a breakdown in immune self‐tolerance, triggering aberrant immune attacks against one's own tissues. These responses cause persistent inflammation and multiorgan damage. As the global prevalence of ADs continues to increase, they impose a growing public health burden, but current treatments do not meet clinical needs. Extracellular vesicles (EVs) are lipid bilayer membrane‐enclosed nanoparticles secreted by live cells that can carry diverse bioactive molecules and play essential roles in intercellular communication. Recently, EVs have attracted considerable attention as promising therapeutic candidates for ADs owing to their high biocompatibility, low immunogenicity, and ability to traverse biological barriers. This review systematically summarizes the current applications and development trends of both plant and mammalian sources and explores the functions of natural or engineered EVs in modulating the pathological processes underlying ADs. We also discuss the emerging potential of EVs as diagnostic biomarkers and targeted drug delivery systems for autoimmune conditions. Although clinical translation of EV‐based therapies faces challenges, deepening our understanding of the pathogenic roles of EVs in autoimmunity coupled with ongoing advances in bioengineering technologies holds promise for delivering novel theoretical insights and practical strategies for diagnosing and treating these refractory diseases.

## Graphical abstract

This review provides a systematic comparison of extracellular vesicles (EVs) from both mammalian and plant sources in the context of autoimmune diseases. It highlights their emerging roles as precision biomarkers and engineered therapeutic platforms. By synthesizing the latest advances and translational challenges, the article offers a roadmap for integrating EVs into next‐generation diagnostic and therapeutic strategies.

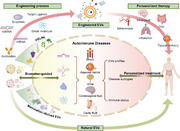

## 1 Introduction

Autoimmune diseases (ADs) are a broad category of chronic disorders characterized by a loss of immune tolerance, resulting in a dysfunctional immune response against self‐antigens and leading to persistent inflammation and tissue damage [1, 2, 3]. The core pathological features of ADs include systemic immune dysregulation, recurrent inflammatory episodes, and progressive organ dysfunction [4]. Common ADs include rheumatoid arthritis (RA), inflammatory bowel disease (IBD), systemic lupus erythematosus (SLE), multiple sclerosis (MS), autoimmune uveitis (AIU), psoriasis (PSO), ankylosing spondylitis (AS), type 1 diabetes mellitus (T1DM), and Sjögren's syndrome (SS) [5, 6]. Epidemiologic studies indicate that ADs collectively affect approximately 10% of the global population, representing a growing public health burden [1, 2, 3]. A large‐scale analysis revealed that the age‐ and sex‐standardized prevalence of 19 major ADs increased significantly from 7.7% to 11.0% between 2000 and 2019. Notably, ADs also demonstrate a notable sex bias, with a higher incidence among women [5]. Although these diseases can occur at any age, some are particularly prevalent among adolescents and young adults, substantially compromising health and quality of life within economically productive segments of the population.

The clinical management of ADs faces several major challenges. First, these diseases often involve multiple organ systems and exhibit high heterogeneity, with diverse pathogenic subtypes [7], making it difficult for existing therapeutics to cover all disease manifestations. Second, their pathogenesis is highly complex and involves breakdowns in immune tolerance, aberrant activation of T and B cells, and persistent proinflammatory signalling [8]. This complexity complicates precise disease classification and targeted treatment. Moreover, the presence of specialized physiological barriers, such as the blood–brain barrier (BBB) and blood–ocular barrier (BOB), restricts the delivery of many immunosuppressive drugs to affected tissues, further limiting therapeutic efficacy [9]. Conventional treatments focus primarily on anti‐inflammatory and immunosuppressive strategies. Although these approaches can partially suppress inflammatory and autoimmune activity, their nonspecific mechanism often leads to substantial side effects, including increased risks of infections, osteoporosis, and hepatic or renal toxicity. Although biological agents such as tumor necrosis factor‐alpha (TNF‐α) inhibitors provide better targeting, their utility is limited by variable patient response rates and the development of drug resistance over time [10]. Therefore, there is a pressing need to develop novel treatment strategies that are more efficient, target specific, and well tolerated.

**FIGURE 1:**
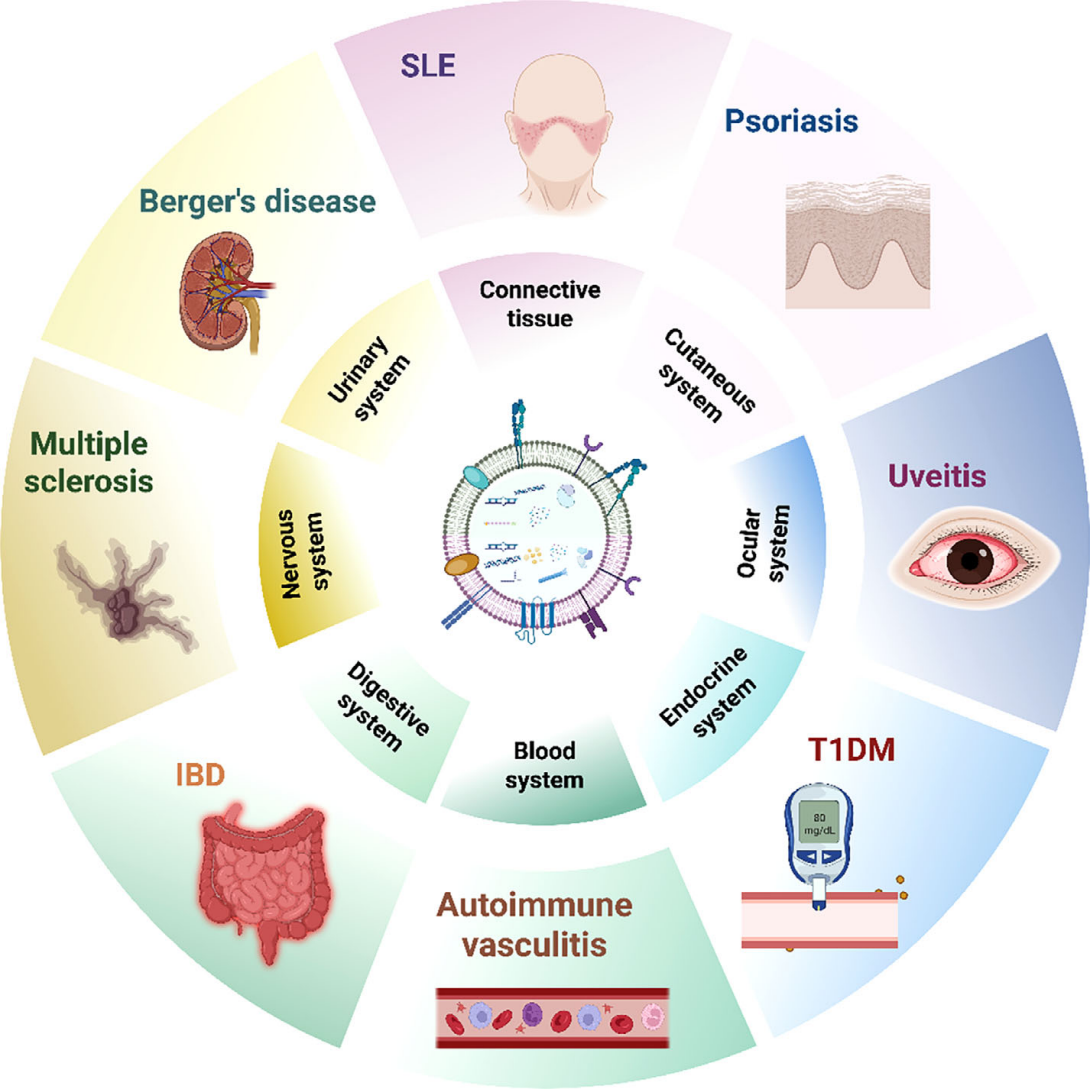
Schematic illustrating the roles of Extracellular Vesicles in various Autoimmune diseases. The central circle depicts the structural diagram of plant‐derived EVs above and mammalian‐derived EVs below. The central circle categorizes ADs on the basis of the primary systems they engage, whereas the outermost circle enumerates individual ADs associated with various systems, demonstrating the functions of EVs across these situations. Abbreviations: EVs, extracellular vesicles; ADs, autoimmune diseases; IBD, inflammatory bowel disease; SL, systemic lupus erythematosus; T1DM, Type 1 diabetes mellitus.

In recent years, extracellular vesicles (EVs) which are nanoscale, cell‐derived lipid vesicles, have attracted increasing interest as promising therapeutic agents in AD research because of their unique biological characteristics (Figure 1) [11, 12, 13]. EVs can carry diverse bioactive molecules (e.g., nucleic acids, proteins, and lipids) and play essential roles in intercellular communication, serving as innate delivery systems [14]. Their natural phospholipid bilayer structure and nanoscale size endow them with low immunogenicity and an increased ability to cross biological barriers such as the BBB and BOB. Furthermore, EVs can be bioengineered into “smart” delivery platforms loaded with anti‐inflammatory factors, small‐molecule drugs, or nucleic acid drugs to target and regulate key pathogenic pathways in ADs. They also possess inherent immunomodulatory properties that can help reshape the inflammatory immune microenvironment [15]. Together, these attributes position EVs as a breakthrough approach for overcoming the limitations of current AD therapies, including poor targeting, limited biodistribution, and systemic toxicity.

This review provides a systematic integration and comparative analysis of advances in both mammalian‐derived and plant‐derived extracellular vesicle‐like nanoparticles (PELNs) and highlights the emerging role of EVs and their engineered derivatives in modulating the central pathological mechanisms of ADs. By simultaneously examining the complementary advantages, shared mechanistic pathways, and distinct challenges of PELNs and mammalian‐derived EVs, this review offers a comprehensive comparative perspective that is currently lacking in the literature. Furthermore, it describes engineering strategies applied to EVs from different sources and explores their potential as novel biomarkers for precision diagnosis and as platforms for targeted therapy. This review also outlines the key challenges in clinical translation and suggests future research directions, thereby providing an informed foundation for advancing EV‐based diagnostics and therapeutics in ADs.

## 2 Biological Properties of Mammalian‐ and Plant‐Derived Extracellular Vesicles

Mammalian‐derived EVs and PELNs exhibit distinct structural and functional characteristics, which directly influence their isolation and purification strategies, bioengineering potential, and biomedical applications in autoimmune diseases; this section systematically elaborates on their structural features, functional differences, key techniques for isolation and purification, and common engineering approaches, laying a foundational framework for understanding their therapeutic mechanisms and translational potential (Table 1).

**TABLE 1:** Key Characteristics and Biomedical Potentials of Plant‐Derived Extracellular Vesicle‐Like Nanoparticles versus Mammalian‐Derived Extracellular Vesicles.

Feature Dimension	Mammalian‐derived EVs	PELNs(Plant‐derived Nanovesicles)	Refs.
Scalability potential	Limited	High	[18]
Immunogenicity	Species‐specific immunogenicity risks present	Naturally low immunogenicity	[17, 19]
Targeting mechanism	Inherent homing capabilities	Passive targeting dominant; enhancement requires surface engineering	[19, 20]
Drug‐loading profile	Compatible with macromolecules (nucleic acids and proteins)	Primarily small molecules; low macromolecule loading efficiency	[21, 22]
Bioactive components	Bidirectional intercellular communication mediators	Rich in polyphenols/flavonoids with potent antioxidant/anti‐inflammatory activities	[18, 23]
Biosafety concerns	Potential viral contamination	Free of zoonotic pathogen risks	[18]
Stability	Moderate; susceptible to inactivation	High	[18]
Manufacturing Barriers	Challenges in industrial‐scale production	Cost‐effective with readily available raw materials	[17]
Primary applications	• Tumor‐targeted therapy • Stem cell regenerative medicine •Anti‐inflammatory/immunomodulatory therapy	• Oral delivery systems (anti‐inflammatory/antioxidant) • Transdermal drug delivery • Nutraceutical development	[18, 19, 24, 25]
Biodistribution	Intravenous administration: mainly distributed in liver, spleen, and inflammatory tissues; some can cross BBB/BOB	Oral administration: primarily enriched in intestinal mucosa and inflamed colonic tissues; transdermal administration: enriched in skin lesions; lower liver/spleen accumulation than mammalian EVs after intravenous injection	[26]
Engineering strategy differences	Easy for genetic/chemical modification with precise targeting; suitable for systemic organ‐targeted therapy due to membrane structure advantages	Relies on chemical encapsulation with limited targeting; optimal for local/oral administration (focused on small‐molecule loading) due to plant‐specific membrane composition (phytosterols and glycosylated proteins)	[21, 26, 27]
Regulatory path	Regulated as cell‐derived products (complies with GMP standards, viral screening, and long‐term safety data; refers to FDA/EMA cell therapy guidelines and MISEV consensus)	Regulated as natural products or nanomedicines (plant raw materials meet food/drug raw material safety standards; no exclusive regulatory framework, refers to nanomedicine and dietary supplement regulations)	[13, 26, 27]

### 2.1 Structural and Functional Characterization

**FIGURE 2:**
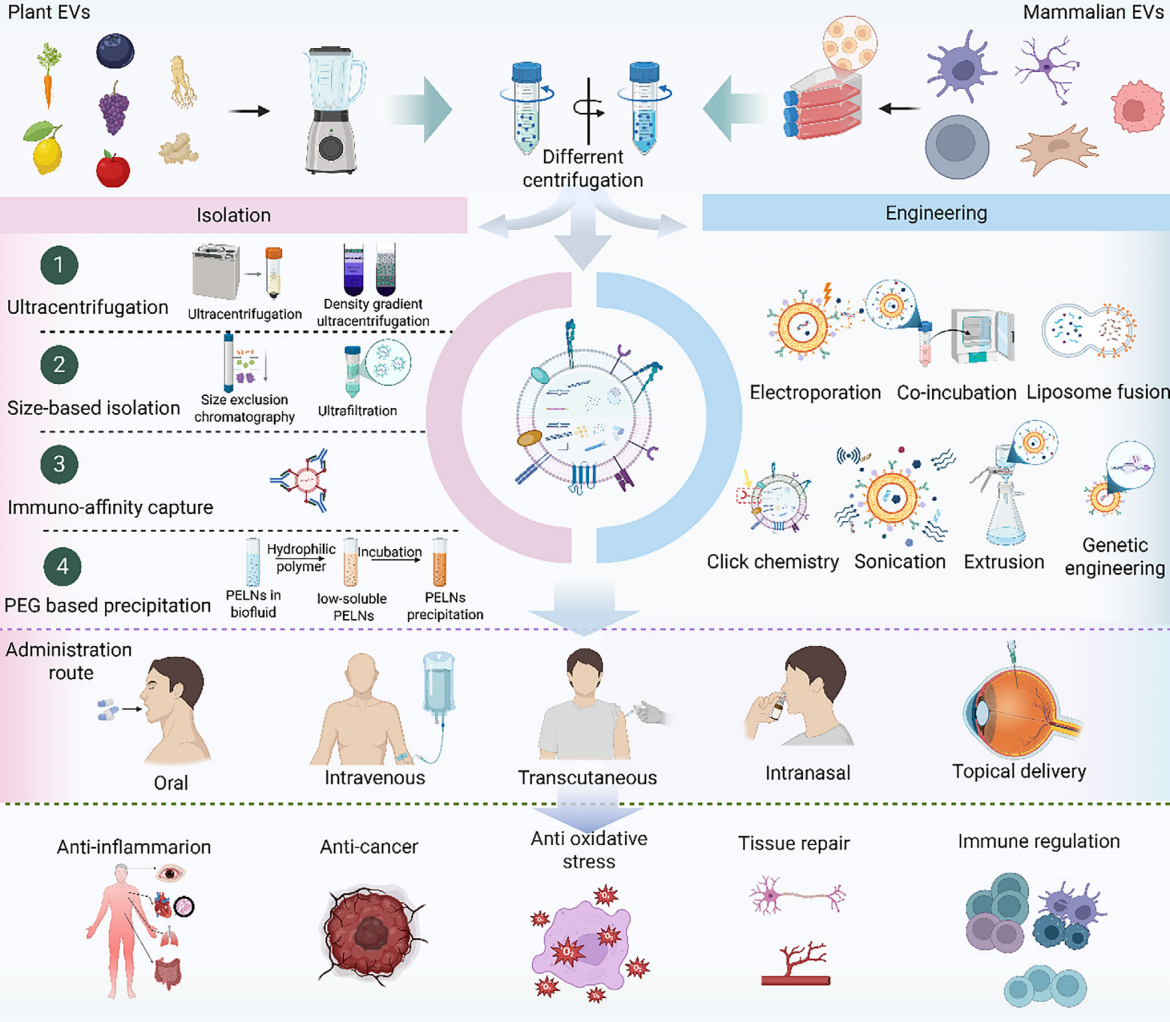
Schematic representation of the isolation and engineering of Extracellular Vesicles. The upper section illustrates the extraction preprocessing of plant‐derived EVs (left) and the synthesis and preprocessing of mammalian‐derived EVs (right). The center layer illustrates strategies for isolating EVs on the left and engineering procedures on the right. The bottom layer delineates the fundamental therapeutic functions of EVs, including anti‐inflammatory, anticancer, antioxidative stress, tissue repair, and immune regulatory activities. **Abbreviations**: EVs, extracellular vesicles; PEG, polyethylene glycol; PELNs, plant‐derived extracellular vesicle‐like nanoparticles.

EVs are nanoscale, membrane‐bound carriers that play central roles in intercellular communication and the regulation of physiological and pathological processes. The main types of EVs that possess anti‐inflammatory and immunomodulatory effects include mammalian‐derived EVs [16] and PELNs [17], whose have been widely recognized for their anti‐inflammatory and immunomodulatory properties are widely recognized. Owing to the significant interspecies differences in structural composition and drug loading, this study provides an in‐depth description of EVs from these two distinct sources (Figure 2).

#### 2.1.1 Mammalian‐Derived Extracellular Vesicles

Mammalian‐derived vesicles are a heterogeneous population of lipid‐bilayer vesicles secreted by human or animal cells and primarily include exosomes, microvesicles, and apoptotic bodies. Typically ranging from 30–150 nm in diameter, these EVs possess a lipid bilayer surface enriched with tetraspanins, integrins and adhesion molecules [14], which serve as cell‐specific “molecular tags” to facilitate targeted cellular recognition and membrane fusion. The lumen contains various bioactive molecules, such as mRNAs, miRNAs, long noncoding RNAs, functional proteins, and metabolites [23]. Cargo loading is selectively regulated by Rab GTPase and RNA‐binding proteins.

Functionally, mammalian‐derived EVs mediate cell‐specific targeting through surface molecules such as integrins and tetraspanins, enabling precise modulation of recipient cell functions via their molecular cargo. Under physiological conditions, they contribute to tissue repair [23] and immune homeostasis by transmitting anti‐inflammatory and pro‐regenerative signals [28]. In pathological contexts, however, their roles are dualistic: certain EVs promote inflammation and disease progression, whereas others suppress inflammatory responses and aberrant immune activation [15, 25]. This functional versatility stems from the dynamic regulation of their membrane composition and cargo sorting, highlighting their potential for precise therapeutic targeting.

#### 2.1.2 Plant‐Derived Extracellular Vesicle‐Like Nanoparticles

PELNs are vesicles ranging from 50–200 nm in diameter that are isolated from fruits, vegetables, and traditional Chinese medicinal herbs. Their membranes are rich in distinct components, such as phytosterols, polyphenols and glycosylated proteins [17], which confer high antioxidant capacity, structural stability, and low immunogenicity. Their internal cargoes include plant‐specific miRNAs, secondary metabolites and antioxidant enzymes [29], enabling cross‐species regulation of physiological functions. Targeted delivery of PELNs is mediated through the interaction between surface glycoproteins and carbohydrate receptors on intestinal epithelial cells, followed by efficient cellular uptake via endocytosis [30]. Functionally, PELNs exert antioxidant and anti‐inflammatory effects through their bioactive contents. For example, catechin‐containing PELNs derived from tea plants have been shown to alleviate oxidative stress in colonic epithelial cells [31]. Moreover, large‐scale agricultural production offers a cost‐effective and sustainable source of PELNs, free from the risk of mammalian‐derived viral contamination [19], underscoring their economic and safety advantages for therapeutic applications in inflammatory and oxidation‐related diseases.

### 2.2 Isolation and Purification of Extracellular Vesicles

EV separation techniques fall into three main categories: physical separation methods, affinity‐based capture methods, and emerging technologies. Ultracentrifugation (UC) is considered the most widely used and historically established method and enriches EVs through multiple centrifugation steps; however, it is time‐consuming and susceptible to the coisolation of contaminants [32]. Size exclusion chromatography (SEC) separates EVs on the basis of differences in gel pore size, preserving their biological activity but often resulting in limited yield (<100 µg/mL). Density gradient centrifugation (DGC) increases purity when using iodixanol gradients are used, although the procedure is relatively labor intensive. Among affinity‐based approaches, immunomagnetic beads achieve high purity (>95%) by targeting specific surface markers (e.g., CD63/CD81); however, the antibodies required are costly and may induce conformational changes in membrane proteins. Lectin affinity capture (e.g., **ConA‐Sepharose**) specifically enriches certain EV subtypes by targeting glycosylated surface motifs [33]. Microfluidic chips enable high‐throughput EV separation within 30 min using fluid dynamics principles, but standardization and scalability remain challenges. EV membranes are rapidly concentrated via filters with 30–200 nm pores, although mechanical stress may cause vesicle deformation [32]. Each approach presents distinct trade‐offs concerning purity, yield, cost, and effects on vesicle integrity, requiring meticulous selection aligned with research objectives, such as the necessity for clinical‐grade purity or high‐throughput processing (Table 2).

**TABLE 2:** Comparative Analysis of Major Techniques for the Isolation and Purification of Extracellular Vesicles.

Isolation Technique	Technical Description	Advantages	Disadvantages	References
Ultracentrifugation	A classical method for isolating exosomes via centrifugation at different speeds, removing impurities based on density differences	Simple operation; relatively high yield	Time‐consuming; prone to protein contamination	[34]
Density gradient centrifugation	Purifies exosomes based on buoyant density differences by adding density gradient medium (e.g., sucrose, iodixanol) to the ultracentrifugation	High purity; reduced impurity contamination	Complex operation; more time‐consuming; low yield	[34]
Ultrafiltration	Separates exosomes through ultrafiltration membranes with specific pore sizes using pressure differences while achieving concentration	Simple and convenient operation	Membrane easily clogged; potential deformation of EVs	[34]
Immunoaffinity capture	Captures exosomes through antigen‐antibody reactions using antibodies against specific surface markers of exosomes (e. g., CD63 and CD81)	High specificity; extremely high purity	High cost; potential alteration of biological functions	[34]
Polymer precipitation	Precipitates exosomes using polymers (e. g., PEG) to reduce their solubility in solution	Simple operation; low cost	Low purity; potential impact on EV activity	[34]
Microfluidic chip	Separates exosomes based on their physical properties (e.g., size and refractive index) using microscale structures in microfluidic chips	High efficiency; short time requirement; good integrity of EVs; high sensitivity	High cost; low popularity; limited large‐scale separation capability	[34]

### 2.3 Engineering Extracellular Vesicles

Engineered EVs refer to EVs that have been structurally or functionally modified through physical, chemical, or biological methods to confer novel biological properties and functions. These modifications are designed to achieve multifunctional integration, thereby increasing their therapeutic potential for disease treatment. Common engineering methods include electroporation, liposome fusion, chemical surface modification, genetic engineering, sonication, extrusion, and incubation.

Electroporation uses an electric field to generate transient pores in the EV membrane, facilitating the diffusion of therapeutic agents into the lumen before pore resealing. This technique is widely utilised for the passive loading of proteins and mRNAs. Notably, electroporation increases the stability and cellular‐targeting efficiency of molecules with inherent instability [35]. Liposome fusion leverages the self‐assembly properties of lipid molecules, thereby incorporating functional groups into EV membranes and increasing vesicle formation and efficient drug loading [36]. Chemical surface modification, often achieved via click chemistry or physical interactions, not only simplifies purification but also promotes receptor‐mediated endocytosis, facilitating cellular uptake and in vivo targeting [12].

Genetic engineering strategies include gene overexpression and silencing, the introduction of exogenous genes and the modification of signal peptides or signal sequences. These techniques enable precise editing of the EV genome, leading to tailored alterations in vesicle composition and function [37]. Sonication uses high‐frequency sound waves to generate transient membrane pores, allowing drug diffusion followed by spontaneous membrane repair. This method is particularly efficient for loading susceptible nucleic acids such as siRNA, improving their in vivo stability and targeting capability [38]. Incubation relies on hydrophobic interactions between drug molecules and the EV lipid bilayer to achieve passive diffusion loading. This straightforward method preserves native targeting proteins and extends the in vivo circulation half‐life of EVs [39]. Extrusion involves the application of mechanical shear to incorporate medicines into the interstices of lipid bilayers, thereby restructuring vesicle architecture. Owing to its elevated drug‐loading capacity (>30%) and ability to codeliver both hydrophilic and hydrophobic chemicals, extrusion is ideally suited for scalable industrial production [40]. Extensive research has demonstrated the broad prospects of engineered EVs for improving the treatment of ADs. Currently, engineered EVs primarily exert their biological functions through three mechanisms: drug loading [41], specific cell targeting [42], and protein expression regulation [43] (Table 3).

**TABLE 3:** Comparative Analysis of Engineering Strategies for Functionalizing Extracellular Vesicles.

Engineering Technique	Technical Description	Targeting Modification Strategy	Advantages	Applicable Precision Medicine Scenarios	Disadvantages	References
Electroporation	Utilizes transient high‐voltage electric fields to create micropores in exosome membranes, allowing target substances to enter	Combines ligand modification for synergistic drug loading and targeting	Efficient loading of macromolecules	Enables on‐demand loading of patient‐specific nucleic acid drugs (e.g., personalized miRNAs) to precisely regulate pathogenic gene expression	Damages EV structures and reduces EV stability	[44, 45]
Liposome fusion	Encapsulates target substances in liposomes, and achieves substance transfer through the fusion of liposomes with exosome membranes	Premodifies liposomes with targeting ligands, retaining targeting activity post‐fusion	Increases targeting of EVs; enables synergistic loading and improves delivery efficiency	Adapts to target differences among ADs subtypes for subtype‐specific therapy	May trigger immune responses; prone to exosome aggregation; high cost	[44, 45]
Chemical surface modification	Uses chemical cross‐linking agents or reactive groups to conjugate targeting molecules to the surfaces of exosomes	Conjugates targeting ligands via click chemistry for precise anchoring to target tissues	Enables precise targeting; good stability	Customizes surface modification based on receptor expression profiles of patient lesions to achieve lesion‐specific enrichment	May affect biological activity of EVs; prone to nonspecific modification	[44, 45]
In vitro co‐incubation	Incubates exosomes with target substances under appropriate conditions and realizes loading through concentration gradient or passive diffusion	Adds targeting ligands during incubation, which adsorb to EV surfaces via hydrophobic interactions	Simple operation	Suitable for adjuvant therapy in patients with mild ADs, enabling rapid adaptation to low‐dose personalized drug delivery	Low loading efficiency; instability; poor specificity	[44, 45]
Sonication	Changes the permeability of exosome membranes through ultrasonic waves of certain frequencies and intensities, promoting the entry of target substances	Modifies with targeting molecules post‐sonication to increase targeting to the central nervous system (CNS)	Relatively high loading efficiency; fast operation and easy scaling up	Enables precise local delivery for focal lesions (e.g., joints and eyes) to reduce systemic side effects	Damages EV structures; poor loading uniformity	[44, 45]
Extrusion	Destroys exosome membranes through mechanical extrusion, forcing drugs to be embedded inside or in membrane gaps, and reshapes the membrane structure	Integrates targeting lipids during extrusion to improve target tissue affinity	High drug loading capacity; wide applicability; fast industrialization	Coloads multiple precision‐targeted drugs (e.g., anti‐inflammatory factors + signalling pathway inhibitors) to adapt to the multitarget pathological characteristics of ADs	Destroys membrane integrity; drug leakage; large batch differences	[44, 45]
Genetic engineering	Modifies parent cells through gene editing, making exosomes express targeting peptides or fusion proteins on their surfaces	Edits parent cells to express disease‐specific targeting molecules, with EVs naturally carrying targeting sites post‐secretion	High targeting; excellent biocompatibility	Customizes EV targeting molecule expression based on patient genetic testing results to achieve "gene‐target‐drug" precise matching	High technical threshold; high cost; prone to triggering immune responses	[44, 45]

## 3 Mechanisms of Extracellular Vesicles in Autoimmune Diseases

**FIGURE 3:**
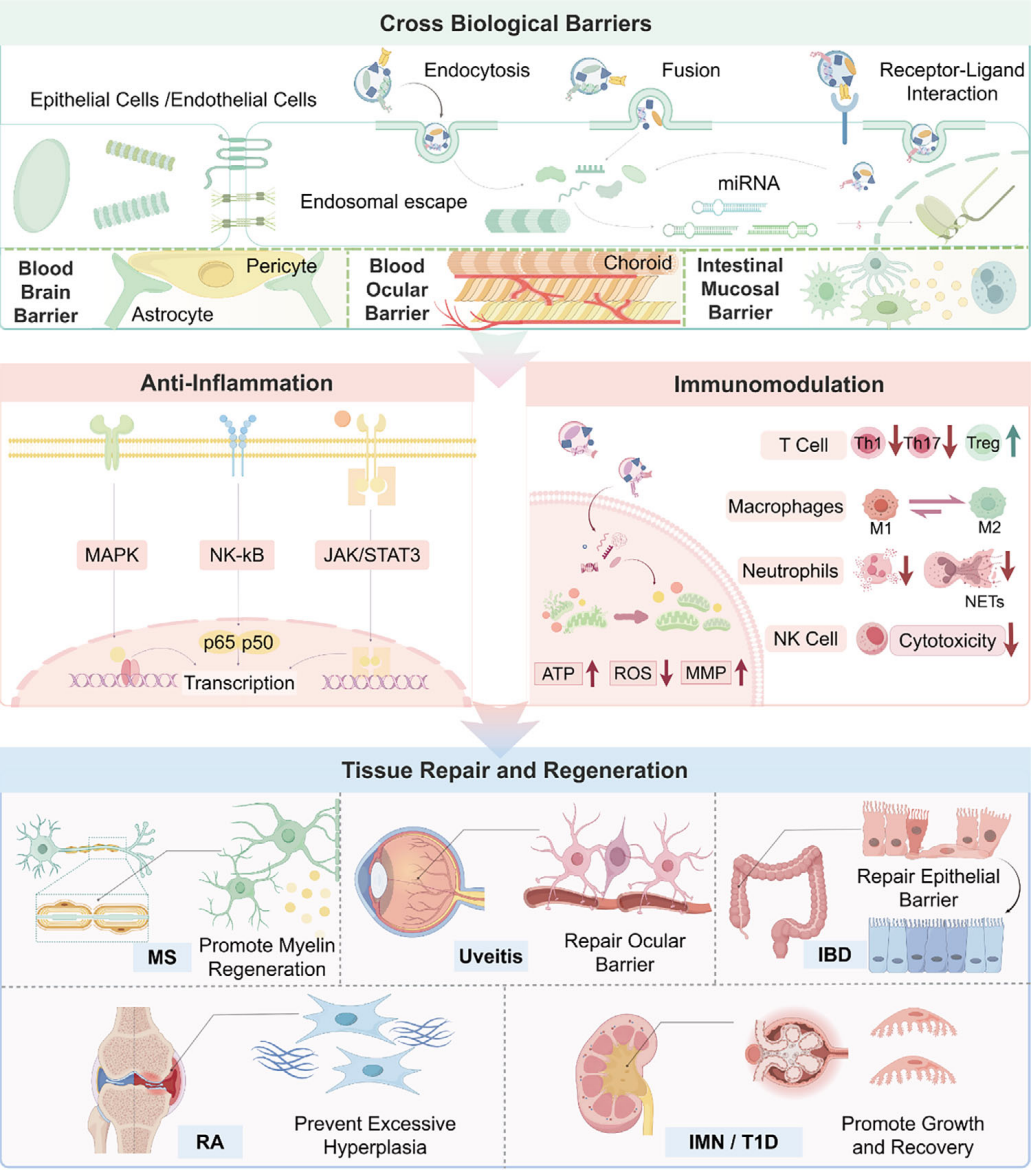
The molecular mechanisms by which extracellular vesicles regulate autoimmune diseases. The initial layer demonstrates that EVs can traverse biological barriers through several mechanisms, including endocytosis, fusion, and receptor–ligand interactions. Key biological barriers include the blood‐brain barrier (BBB), blood‐ocular barrier (BOB) and the intestinal mucosal barrier. The second layer shows illustrates that EVs participate in the control of some traditional inflammatory pathways (left) and the reconstruction of the immune microenvironment (right). EVs modulate mitochondrial function by transporting bioactive molecules (e.g., miRNAs and proteins), thereby affecting the development and secretion of immune cells such as T cells and macrophages. The lowest layer signifies EVs, which significantly contribute to tissue repair and regeneration in a bidirectional manner, from inhibiting excessive hyperplasia in RA to repairing the epithelial barrier in IBD. Abbreviations: ATP, adenosine triphosphate; ROS, reactive oxygen species; MMP, mitochondrial membrane potential; GC, germinal center; PC, plasma cell; NETs, neutrophil extracellular traps; RA, rheumatoid arthritis; IMN, idiopathic membranous nephropathy; T1D, type I diabetes; IBD, inflammatory bowel disease.

The mechanisms through which EVs modulate ADs involve several key biological processes, such as transporting bioactive molecules across biological barriers, modulating critical inflammatory signalling pathways, regulating immune cell activities, remodeling the immune microenvironment, and participating in tissue repair and regeneration. The following sections elaborate on these mechanisms in detail (Figure 3).

### 3.1 Cross‐Biological Barrier Delivery

Owing to their nanoscale size, natural lipid bilayer structure, and low immunogenicity, EVs can effectively penetrate biological barriers and efficiently deliver therapeutic drugs to localized lesions. This capability provides a distinct advantage for treating neurological and ocular diseases. However, the mechanisms of barrier penetration vary considerably depending on the cellular origin of the EVs.

EVs cross barriers such as the BBB primarily via receptor‐mediated interactions with endothelial cells. For instance, microglia‐derived EVs adhere to endothelial cells through surface integrins. This interaction activates Src kinase, mediating the phosphorylation of tight junction proteins, which transiently opens the BBB and facilitates transmigration [46]. Engineered EVs can increase targeting specificity, for example, EVs expressing rabies virus glycoprotein (RVG) bind to nicotinic acetylcholine receptors (nAchRs) on neurons, enabling precise drug delivery to the brain [47]. As nanocarriers, EVs have also been shown to improve drug permeability across the BOB. Retinal pigment epithelial cell‐derived EVs can bind to Gas6 ligands via MERTK receptors on the cell membrane. These EVs traverse the outer barrier through phagocytosis and penetrate the inner barrier via interactions between CD44 and HA. Importantly, EVs improve drug penetration, prolong ocular tissue contact time, enable site‐specific delivery, sustain drug release, and reduce systemic toxicity and side effects [20]. For instance, adipose‐derived MSC‐derived EVs enriched with microRNA‐24‐3p formed a uniform layer on the corneal surface, prolonging drug‒epithelium contact time and promoting corneal epithelial defect healing [48].

PELNs primarily rely on their natural glycosylations and membrane lipid components to cross barriers. In the case of the intestinal mucosal barrier, for example, PELNs from grapefruit bind to lectin receptors on intestinal epithelial cells via surface arabinogalactan proteins. They then enter the circulatory system via clathrin‐mediated endocytosis and evade the first‐pass effect of the liver via the lymphatic pathway [49]. Additionally, plant sterols can interact with cholesterol in BBB endothelial cell membranes, altering membrane fluidity to facilitate fusion and increase the efficiency of drug delivery to the brain [18].

### 3.2 Anti‐Inflammatory Regulation

EVs can play an immunoregulatory role in ADs by targeting key inflammatory signalling pathways. This section outlines three major inflammatory pathways frequently implicated in EV‐mediated regulation: the NF‐κB, MAPK, and JAK–STAT pathways. The NF‐κB pathway is central to immune and inflammatory responses and regulates the expression of multiple proinflammatory factors and the activation of inflammasomes [50]. For example, EVs derived from gingival MSCs alleviate collagen‐induced arthritis (CIA) by inhibiting the IL‐17RA–Act1–TRAF6–NF‐κB signalling cascade [51]. The MAPK pathway also plays a critical role in innate immune responses, participating in both proinflammatory responses and anti‐inflammatory feedback mechanisms [52]. PELNs from ginseng attenuate IBD by counteracting the LPS‐induced upregulation of MAPK pathway proteins, thereby exerting anti‐inflammatory effects [53]. The JAK–STAT pathway is associated with various ADs [54]. EVs derived from human umbilical cord MSCs (hUCMSC‐EVs) ameliorate lupus nephritis (LN) by increasing renal STAT3 activation and reducing IL‐17A protein levels [55]. Notably, EV‐mediated modulation of JAK–STAT signalling is not uniformly inhibitory; in some contexts, it involves activation. For example, olfactory ecto‐MSC‐derived EVs (OE‐MSC‐EVs) secrete IL‐6, which activates the JAK2–STAT3 pathway in myeloid‐derived suppressor cells (MDSCs), increasing their immunosuppressive function and alleviating disease [56]. In addition to being involved in intracellular signalling, EVs are involved in complement activation pathways. Autologous EVs have been shown to drive immune dysfunction in immune thrombocytopenia via the lectin pathway and the resistin–myeloid factor–adipokine metabolic axis, promoting disease onset [57]. Conversely, ageing neutrophil‐derived EVs can exert sustained anti‐inflammatory effects by displaying “do not eat me” signals that evade phagocytosis and accumulate during inflammation resolution. Surface CD55 inhibits complement C3 convertase activity, preventing complement cascade activation and contributing to robust anti‐inflammatory activity [58].

### 3.3 Immunomodulation

The immune microenvironment comprises a highly organised network of interacting cellular components that drive inflammatory processes. Through dynamic multicomponent interplay, this system disrupts immune homeostasis, creating a self‐amplifying loop of inflammation and tissue injury that is the core driver of AD pathogenesis. EVs act as critical mediators of this process. By engaging with immune cells, EVs actively remodel the inflammatory microenvironment, thereby exerting bidirectional control over disease progression, either ameliorating or exacerbating pathological outcomes.

MicroRNA (miRNA)‐mediated regulation represents a pivotal mechanism underlying the function of EVs. As carriers of diverse molecular cargoes, including miRNAs, lncRNAs, tsRNAs, proteins and lipids [59], EVs play a critical role in modulating intercellular communication. Notably, miRNAs contribute significantly to immune cell reprogramming in ADs, highlighting their diagnostic and therapeutic potential [60]. For instance, miRNA‐146a enriched in MSC‐EVs targets the inflammatory genes TRAF6 and IRAK1, thereby suppressing their expression and inhibiting NF‐κB pathway activation. This mechanism effectively ameliorates trinitrobenzene sulfonic acid (TNBS)‐induced colitis in rats [61]. Similarly, Cai X et al. reported that MSC‐EVs can bind directly to NLRP3 mRNA via miR‐410, inhibiting the NLRP3 pathway and reducing NLRP3 inflammasome activation in macrophages [62]. In MS, MSC‐EVs carrying anti‐inflammatory miRNAs such as miR‐125b significantly reduce proinflammatory cytokine secretion by peripheral blood mononuclear cells (PBMCs), thereby restoring immune imbalance [13]. Conversely, EVs produced under pathological conditions can actively contribute to disease pathogenesis. In antiphospholipid syndrome (APS), for example, elevated levels of EVs carrying bioactive lipids, proteins, and nucleic acids (particularly miRNAs) promote thrombus formation and vascular activation, thereby exacerbating the disease [63]. Notably, astrocyte‐derived EVs (AST‐EVs) deliver miR‐129‐2‐3p to oligodendrocytes and optic nerve tissue, activating SMAD3 target genes and inducing demyelination lesions [64]. These findings suggest that EVs could serve as specific biomarkers for the diagnosis of neuromyelitis optica (NMO) and represent promising therapeutic targets.

In addition to affecting the miRNA‐mediated reprogramming of immune cells [65], EVs also modulate the inflammatory microenvironment by regulating mitochondrial function and energy metabolism in immune cells [66]. Growing evidence indicates that EVs influence metabolic pathways through multiple mechanisms. For example, PELNs from ginseng reverse LPS‐induced decreases in mitochondrial membrane potential (MMP) and macrophage abundance, reduce reactive oxygen species (ROS) production, and alleviate symptoms in IBD [53]. When administered to chondrocytes, PELNs from *Spirulina platensis* mitigate osteoarthritis (OA) progression by restoring the MMP, ameliorating mitochondrial dysfunction, replenishing ATP levels, and promoting cartilage matrix synthesis and metabolic homeostasis in chondrocytes. Additionally, adipose‐derived MSC‐derived EVs transfer mitochondrial components to alveolar macrophages, increasing mitochondrial activity and oxidative phosphorylation capacity. This transfer improves metabolic and immune balance, reduces lung injury, and supports tissue repair [67].

Immune cells play pivotal yet complex roles in ADs, functioning as initiators and effectors of inflammatory cascades and directly contributing to tissue pathology. EVs can modulate a broad spectrum of immune cells, with current research on ADs focusing primarily on T cells and macrophages. T lymphocytes are central drivers of AD pathogenesis through self‐antigen recognition, inflammatory activation, direct cytolysis, and the coordination of immune responses. EVs regulate pathogenic T‐cell activities via multiple mechanisms. For instance, bone marrow MSC‐derived EVs (BMSC‐EVs), alleviate RA by suppressing the proliferation of T and B lymphocytes and dose‐dependently inducing Tregs and IL‐10^+^ regulatory B cells [68]. Beyond RA, interferon‐gamma‐stimulated MSC‐EVs (IFN‐γ‐EVs) increase the population of CD4^+^CD25^+^FOXP3^+^ Tregs in the spinal cords of experimental autoimmune encephalomyelitis (EAE) mice, demonstrating notable neuroprotective effects [13]. Similarly, in LN, engineered human umbilical cord MSC‐EVs (hUCMSC‐EVs) rebalance Th1/Th17/Treg ratios and suppress pathogenic double‐negative T cells (DNTs) and plasma cells [55]. In addition, Bolandi et al. confirmed that EVs derived from adipose tissue‐derived MSCs can effectively regulate immune cells and fibroblast‐like synovial cells (FLSs) by suppressing proinflammatory subsets of CD4+ T cells and inducing anti‐inflammatory subsets. This process involves the transfer of specific miRNAs and has potential applications in the treatment of RA [69]. Further studies revealed that programmed cell death protein 1 (PD‐L1), which is present on the surface of EVs, can effectively inhibit follicular helper T (Tfh) cell polarization by suppressing the PI3K/AKT pathway. Furthermore, EVs encapsulated in light‐crosslinked silk fibroin hydrogels derived from olfactory MSCs (EVs@SFMA) can inhibit the differentiation of germinal center B cells into plasma cells even more effectively, thereby alleviating synovial inflammation and joint damage [70].

Innate immune cells, such as macrophages, neutrophils, dendritic cells, mast cells, and innate lymphoid cells (ILCs), play fundamental roles in ADs. Among these, macrophages represent key cellular targets for EV‐mediated immunomodulation. Macrophages exhibit remarkable plasticity, allowing them to polarize into distinct functional phenotypes in response to local microenvironmental cues. In ADs, they can exacerbate inflammation and contribute to tissue injury but also participate in repair processes. On the basis of their polarization status, macrophages can be broadly categorized into proinflammatory (M1) [71] or anti‐inflammatory (M2) [72] subsets, which functionally counterbalance each other to maintain immune homeostasis. Disruption of this equilibrium is closely linked to the initiation and progression of ADs.

Recent evidence has indicated that ginger‐derived EVs alleviate IBD by modulating macrophage M1/M2 polarization via the IKK/IκB/NF‐κB pathway [73]. These vesicles also activate autophagy, facilitating the degradation of cellular components and thereby restoring metabolic and intestinal immune homeostasis [74]. In a related vein, Takenaka et al. demonstrated that EVs loaded with IL‐4 promote M2 polarization, offering therapeutic benefits in patients with RA [75]. To further support the role of EV‐guided macrophage reprogramming, Zhang M. et al. reported that BMSC‐EVs stimulate anti‐inflammatory macrophage polarization in LN models. This effect was accompanied by Treg recruitment mediated through the delivery of miR‐16 and miR‐21, collectively ameliorating LN pathology [76].

In addition to macrophages, neutrophil extracellular traps (NETs) have emerged as critical players in AD pathogenesis [77]. Prolonged NETs exposure to NETs increases the risk of systemic organ damage [78]. In adult‐onset Still's disease (AOSD), miRNA‐223 packaged within neutrophil‐derived small EVs suppresses interleukin (IL)‐18 expression in macrophages, thereby curbing IL‐18‐driven NET formation and attenuating inflammation and tissue injury [79]. Interestingly, M2 macrophage‐derived EVs confer protection in non‐AD contexts, such as sepsis‐induced acute lung injury by limiting neutrophil infiltration and NETosis [80]. Similarly, intravenous administration of MSC‐EVs carrying miRNA‐125a‐3p suppresses NET formation, mitigating spinal cord injury [81]. The immunoregulatory influence of EVs extends to other innate and adaptive immune cells. For instance, Kordelas et al. revealed that MSC‐EVs reduce NK cell cytotoxicity in graft‐versus‐host disease (GVHD) by dampening IFN‐γ and TNF‐α release from activated NK cells [82]. These MDSCs heterogeneous immature myeloid cells with potent immunosuppressive activity [83] extend therapeutic potential through their EVs. Rapamycin‐conditioned MDSC‐EVs (Rapa‐EVs) alleviate GVHD through miR‐181d‐5p‐mediated knockdown of KLF6 [84], whereas G‐MDSC‐EVs suppress Th1/Th17 responses in autoimmune arthritis [85]. Moreover, MDSC‐EVs delivering immunosuppressive miRNAs (e.g., hsa‐miR‐142‐5p and miR‐19a‐3p) to activated T cells ameliorated immune‐mediated bone marrow failure (BMF) [86].

### 3.4 Tissue Repair and Regeneration

EVs promote tissue repair and functional recovery through multiple mechanisms, including stimulating cell proliferation and facilitating extracellular matrix remodeling. In idiopathic membranous nephropathy (IMN), EVs have demonstrated therapeutic potential by increasing glomerular endothelial cell proliferation and supporting the recovery of injured podocytes [87]. Similarly, He et al. reported that EVs derived from bone marrow MSCs (BMSCs) significantly increased cartilage regeneration in a rat model of osteoarthritis [88]. In a more advanced approach, Yang et al. developed a ginsenoside CK‐hybridized EV system (HyExo@CK) incorporated into an injectable microporous hydrogel scaffold (HyExo@CK/SiCH), achieving multidimensional cartilage repair through superior recruitment of endogenous BMSCs and promotion of complete cartilage regeneration [89]. In the skin, Kim et al. demonstrated that canine adipose tissue‐derived EVs (cASC‐EVs) accelerated skin barrier repair in atopic dermatitis by improving stratum corneum hydration and upregulating expression of epidermal differentiation proteins [90]. In neurological contexts, microglia‐derived EVs have been shown to regulate myelin regeneration via activation of the Nrf2 pathway in oligodendrocyte precursor cells, suggesting their utility in demyelinating diseases such as MS [91]. Moreover, EVs derived from human induced pluripotent stem cell‐differentiated neural progenitor cells can promote neuronal survival and suppress microglial overactivation [92]. Given that excessive microglial activation leads to glial scar formation, which impedes neural repair, EVs represent a promising therapeutic avenue for neurodegenerative disorders.

Our team has conducted extensive research on the role of glial cell‐derived EVs in optic nerve injury repair. A series of studies confirmed that EVs derived from Schwann cells [23], astrocytes [93], and Müller glial cells [28] elicit distinct neurorestorative effects, highlighting their potential for the personalized treatment of optic nerve injury and neurodegenerative conditions. Beyond mammalian‐derived EVs, PELNs also exhibit considerable potential in tissue regeneration [94]. For instance, ginseng‐derived EVs were demonstrated to stimulate the neural differentiation of BMSCs through the delivery of incorporated miRNAs [95], whereas ginger‐derived EVs accelerated intestinal mucosa repair [96]. Recently, the integration of the MSC secretome with biomaterial scaffolds, such as hydrogels, electrospun nanofibers, and 3D‐printed constructs, has emerged as a promising strategy in regenerative medicine [97]. These hybrid systems enable the spatiotemporally controlled release of bioactive factors, thereby increasing the efficiency of tissue repair and regeneration.

In addition to promoting tissue repair, EVs can mitigate autoimmune disease by inhibiting programmed cell death pathways such as apoptosis and pyroptosis. For instance, in a type 1 diabetes model, intravenous administration of USC‐EVs was shown to suppress apoptosis in podocytes and renal tubular epithelial cells while simultaneously stimulating the proliferation of glomerular endothelial cells, thereby attenuating diabetic renal complications [98]. Furthermore, EVs can modulate synovial fibroblast behavior in patients with RA. Upon internalization by these fibroblasts, EV‐carried constituents target proliferation‐associated genes, thereby inhibiting fibroblast activation [99]. This suppression helps prevent pathological synovial hyperplasia, aberrant angiogenesis, and the subsequent destruction of joint cartilage and bone processes that collectively drive disease progression [100].

## 4 Studies of the Application of Extracellular Vesicles in Autoimmune Diseases

**FIGURE 4:**
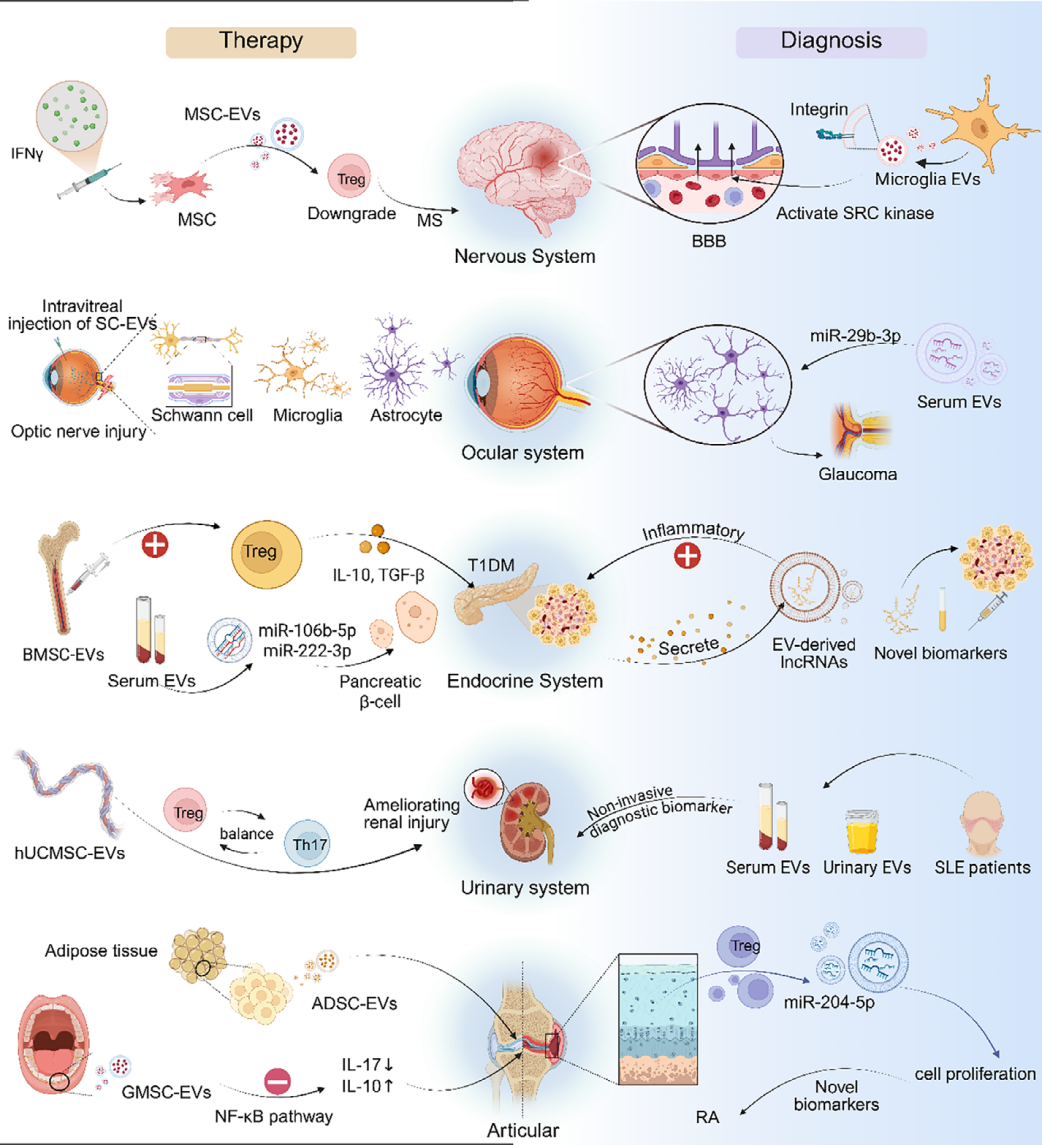
Overview of Extracellular vesicles Applications in Autoimmune diseases. The left side shows therapeutic strategies, the right side shows diagnostic approaches and the middle section is organised by body system (nervous, ocular, endocrine, urinary and articular). **Abbreviations**: EVs, extracellular vesicles; MSC, mesenchymal stem cell; Treg, regulatory T cell; MS, multiple sclerosis; BBB, blood–brain barrier; AST‐EVs, astrocyte‐derived EVs; NMOSDs, neuromyelitis optica spectrum disorders; T1DM, type 1 diabetes mellitus; lncRNAs, long noncoding RNAs; hUCMSC, human umbilical cord mesenchymal stem cell; SLE, systemic lupus erythematosus; GMSC, gingival mesenchymal stem cell; ADSC, adipose‐derived stem cell; RA, rheumatoid arthritis; IL, interleukin; TGF‐β, transforming growth factor‐β; miR, microRNA; BMSC, bone marrow mesenchymal stem cell.

EVs have garnered increasing attention because of their value in the diagnosis and treatment of various ADs owing to their distinctive biological properties and diverse sources (Figure 4). As diagnostic tools, EVs serve as readily accessible, noninvasive biomarkers, offering a practical alternative to conventional detection methods. Therapeutically, EVs exhibit favourable biocompatibility alongside notable anti‐inflammatory and immunoregulatory effects. Moreover, EVs possess intrinsic therapeutic activity and can be flexibly engineered as versatile carriers, positioning them as highly promising platforms for AD treatment. Collectively, these attributes establish a strong foundation for advancing the application of EVs in precision medicine for ADs.

### 4.1 Diagnostic Biomarkers

Molecules carried by EVs, such as nucleic acids and proteins, exhibit disease‐specific characteristics, providing a novel noninvasive tool for the precise diagnosis and dynamic monitoring of ADs. Representative applications include the following: In the development of SLE, the miRNA profile of urinary EVs has been identified as a key indicator of renal involvement [101]. With respect to RA, Wu et al. reported that miR‐204‐5p, an EV‐associated miRNA secreted by T cells, is highly enriched in the synovial fluid of patients with RA and suppresses cell proliferation, highlighting its potential as a diagnostic biomarker [99]. In primary SS, Finamore et al. used SWATH‐MS to characterize the EV subproteome in saliva and identified differentially expressed proteins that may serve as diagnostic markers for pSS [102]. In IBD, label‐free LC‒MS/MS proteomic analysis of serum EVs revealed elevated levels of pregnancy zone protein (PZP), supporting its potential as an auxiliary diagnostic biomarker for IBD [103]. Furthermore, tear‐derived EVs present a convenient, noninvasive source of biomarkers for the early detection and monitoring of ocular complications such as diabetic retinopathy [104]. In the clinical diagnosis of RA, the presence of chondroitin sulfate protein on the surface of EVs in the synovial fluid of patients with knee osteoarthritis (OA) [105], as well as the expression of the synovial homing receptor αVβ3 integrin in plasma EVs [106], can be used as key surface markers. These markers provide an important basis for the accurate, stratified diagnosis of RA.

### 4.2 Therapeutic Applications of Mammalian‐Derived Extracellular Vesicles

EVs possess several advantageous properties, such as potent immunomodulatory effects, innate targeting ability, high biocompatibility, low immunogenicity, and the ability to cross biological barriers, making them promising candidates for the treatment of ADs. They are increasingly regarded as innovative vehicles for drug delivery and therapeutic intervention. Unlike conventional therapies, EVs can deliver diverse cargoes, including proteins, nucleic acids, and small‐molecule drugs, directly to immune cells or affected tissues. The administration route is a key determinant of the biodistribution and efficacy of EVs. As evidenced by studies on oral [107], intravenous [81], intraperitoneal [108], intranasal [109], and topical [110] delivery methods, each method directs EVs to specific tissues and organs, where they elicit their intended effects. Additionally, compared with traditional medicines, EVs can directly influence aberrant immune responses by transporting intrinsic bioactive molecules, which may lead to less harmful and more effective therapeutic outcomes. This section systematically elaborates on the specific therapeutic strategies, mechanisms, and recent advances in EV‐based treatments for several representative ADs (Table 4).

**TABLE 4:** Comprehensive Subtyped Extracellular Vesicles (EVs) for Therapy of Autoimmune Diseases (ADs): Key Characteristics, Advantages, and Applicable Scenarios.

Source of EVs	Subtype	Key Characteristics	Advantages	Engineering Strategies	Applicable Autoimmune Diseases	Reference
Mesenchymal stem cell (MSCs)	Bone marrow MSCs (BMSCs)	Regulates macrophage polarization via NF‐κB; enriched in integrins/CD9/CD63, miR‐16/miR‐21, Arg‐1/CCL20	Potent macrophage plasticity modulation; prominent tissue repair; superior renal tropism	Genetic engineering to overexpress integrin α3β1, electroporation to load miR‐16/miR‐21	SLE, LN, RA, IBD, AIU	[68, 76, 88]
	Human umbilical cCord MSCs (hUCMSCs)	Low immunogenicity; miR‐21‐5p/TGF‐β1‐enriched; modulates Treg/Th17, suppresses DNTs	Broad immunomodulatory spectrum; plasma cell inhibition; scalable manufacturing	In vitro co‐incubation to naturally encapsulate TGF‐β1, no additional modification	SLE, LN, SS, T1DM, POI	[55, 111, 112]
	Gingival MSCs (GMSCs)	High CD44 expression; anti‐inflammatory miRNAs; targets IL‐17RA–Act1–TRAF6–NF‐κB	Potent anti‐inflammation; bone protection; clinical waste‐derived	Liposome fusion to load IL‐17 neutralizing peptide, chemical surface modification to conjugate RGD peptide	RA, IBD	[51, 113]
	Adipose‐derived MSCs (ADSCs)	Adiponectin/miR‐10a‐rich; high membrane stability; inherent immunomodulatory lipids	Abundant adipose source; minimal immunogenicity; transdermal/oral compatible	Sonication to load adiponectin/miR‐10a, chemical surface modification with cholesterol to increase transdermal penetration	RA, atopic dermatitis, PSO	[69, 82, 114]
	iPSC‐derived MSCs (iPSC‐MSCs)	let‐7b‐5p/miR‐21‐5p‐enriched; immunomodulatory plasticity; unlimited proliferation	Rare AD customization; donor‐independent; robust immunoregulation	Genetic engineering to knockout immune rejection gene, CAR gene editing	SS, MS	[111, 115]
	Olfactory Ecto‐MSCs (OE‐MSCs)	Secretes IL‐6/PD‐L1; modulates MDSCs; BBB‐penetrating motifs	Superior neural tropism; efficient BBB crossing; potent neuroprotection	Electroporation to load BDNF, chemical surface modification with dual RVG/TAT peptides	SS, MS, NMOSDs	[56, 70]
Immune Cells	M2‐polarised macrophages (M2‐Mφ)	CD206high; IL‐4/IL‐10‐enriched; inhibits NF‐κB inflammation	Potent anti‐inflammatory polarization; high inflamed foci enrichment; minimal off‐target effects	Endocytosis to load glucocorticoid prodrug, genetic engineering to overexpress CCR2 receptor	RA, IBD, AIH	[75]
	Induced Regulatory T‐cells (iTreg)	Expresses FOXP3/CD25; miR‐449a‐loaded; targets Notch1; modulates Th17/Treg	Pathogenic T‐cells ‐specific regulation; no immune rejection; immune tolerance enhancement	Genetic engineering to stably express FOXP3, chemical surface modification to covalently conjugate CTLA‐4‐Ig	RA, SLE, MS	[116]
	Granulocytic MDSCs (G‐MDSCs)	PGE2/miR‐181d‐5p‐enriched; inhibits T‐ cell activation, Th1/Th17 responses	Broad immunosuppression; multi‐subtype AD alleviation; combination therapy‐compatible	Liposome fusion to load PD‐L1 plasmid, chemical surface modification with PEGylation	RA, GVHD, BMF	[84, 85, 117]
	Neutrophils	Carry miR‐223; expresses CD55; inhibits complement activation, NETs formation	Inflammation resolution; IL‐18‐driven pathology suppression; tissue repair promotion	Intracellular microinjection to load DNaseI, genetic engineering for recombinant CD59 expression	AOSD, IBD	[58, 79]
Stem Cells	Urine‐derived stem cell (USCs)	Noninvasive access; miR‐106b‐5p/miR‐222‐3p‐enriched; anti‐apoptotic proteins; renal affinity	Exceptional renal tropism; low ethical concerns; cost‐effective scalability	Sonication to load anti‐fibrotic drug, chemical surface modification to conjugate nephrin‐binding peptide	T1DM, LN, IMN	[118]
	Umbilical cord blood MNCs (UCB‐MNCs)	Anti‐inflammatory miRNAs; induces M2 polarization; modulates DC function	Low immunogenicity; abundant source; suitable for pediatric AD; immune tolerance enhancement	Lentivirus‐mediated gene transfection to load IL‐10 gene	PSO, atopic dermatitis	[119]
Tissue Cells	Corneal cells (epithelial/stromal)	Expresses CD44/HA receptors; efficient BOB penetration; carry neuroprotective factors	Excellent ocular tropism; minimal ocular irritation; corneal repair promotion	Liposome fusion to load lactoferrin nanoparticle, genetic engineering for recombinant hyaluronic acid‐binding domain expression	AIU, SS‐associated dry eye, TED	[20, 48, 120]
	Retinal pigment epithelial (RPE) cells	Expresses MERTK; neuroprotective cytokines; reinforces blood‐retinal barrier	Superior retinal targeting; ocular tissue repair; intraocular inflammation modulation	Electroporation to load VEGF inhibitor, genetic engineering to overexpress CD47	AIU, NMOSDs, glaucoma	[20, 120]
	Schwann cells	Carry neurotrophic factors; regulates myelin regeneration; neural cell‐adhesive molecules	Prominent neural tropism; optic nerve repair; neuroinflammation modulation	In vitro coincubation to naturally load NGF, chemical surface modification to conjugate MBP‐binding peptide	MS, NMOSDs	[91, 121]
	Astrocytes	miR‐129‐2‐3p‐loaded; regulates neuroinflammation; interacts with microglia	Strong CNS tropism; BBB penetration facilitation; demyelination suppression	Liposome fusion to load miR‐124, chemical surface modification to conjugate CD11b ligand	MS, NMOSDs	[64, 121]
	Müller glia	Neuroprotective miRNAs; inhibits microglial overactivation; promotes retinal survival	Exceptional retinal tropism; suitable for ocular neurodegenerative AD‐suitable; visual function preservation	Genetic engineering to transfect Bcl‐2 anti‐apoptotic gene, chemical surface modification to conjugate Syntaxin‐binding domain	AIU, glaucoma, optic nerve injury	[28, 93]
	Renal tubular epithelial (HK‐2) cells	Carry anti‐inflammatory factors; inhibits NF‐κB/NLRP3; reinforces renal barrier	High kidney specificity; renal inflammation alleviation; podocyte protection	Electroporation to load NLRP3 inhibitor, chemical surface modification to conjugate megalin receptor ligand	IgAN, LN	[118]
	Pancreatic β‐cells	β‐cell regeneration miRNAs; modulates islet inflammation; increase β‐cell survival	Superior islet tropism; β‐cell function protection; autoimmune diabetes alleviation	In vitro co‐incubation to naturally load GLP‐1, genetic engineering to overexpress PD‐L1	T1DM	[122, 123]
Plant‐derived (PELNs)	Ginseng	Ginsenosides/polyphenols‐rich; plant miRNAs; targets TLR4/MAPK	Dual anti‐inflammatory/antioxidative; excellent oral stability; gut‐immune axis modulation	Sonication to load ginsenosides and miRNAs, chemical surface modification with chitosan coating	RA, IBD	[53, 124]
	Ginger	Curcumin/phytosterols‐enriched; regulates macrophage polarization; activates autophagy	Superior intestinal tropism; intestinal homeostasis repair; gut barrier reinforcement	Cyclodextrin inclusion to improve curcumin solubility, chemical surface modification to conjugate mannose	IBD	[73, 96]
	*Portulaca oleracea*	Flavonoids/antioxidant enzymes; promotes CD4^+^CD8^+^T cell expansion; modulates gut microbiota	Accessible raw materials; cost‐effective scalability; no zoonotic risk	/	IBD	[125]
	Grapefruit	Surface arabinogalactan proteins; targets intestinal macrophages; upregulates HO‐1 expression	Inflamed colon enrichment; proinflammatory cytokine suppression; good oral tolerance	Sonication to load anti‐inflammatory components, chemical surface modification to conjugate TNF‐α antibody fragment	IBD	[30]
	Broccoli	Sulforaphane‐rich; activates AMPK; inhibits DC overactivation	Synergistic anti‐inflammatory/immunomodulatory; intestinal epithelial repair	/	IBD	[126]
	Grape	Unique membrane lipids; promotes Lgr5^+^ intestinal stem cell proliferation; increases organoid formation	Potent mucosal repair; gut regeneration acceleration; gut barrier reinforcement	Solvent extraction to isolate active components, chemical surface modification to conjugate Lgr5 ligand	IBD	[127]
	Turmeric	Curcumin‐enriched; inhibits NF‐κB; modulates inflammatory genes	Potent anti‐inflammation; high oral bioavailability; skin/gut AD‐suitable	Liposome fusion for skin use, enteric capsule coating for intestinal use	IBD, PSO	[128]
	Tea plant	Catechins‐rich; alleviates oxidative stress; modulates epithelial redox balance	Prominent antioxidation; targets inflammation‐related oxidative damage; oral‐compatible	Chemical surface modification with vitamin C synergistic encapsulation to stabilize catechins	IBD, atopic dermatitis	[19, 114]
Milk‐derived	Bovine milk‐derived EVs	Whey protein/lactoferrin/sphingolipids; nonimmunogenic; gastrointestinal‐tolerant	High oral bioavailability; superior intestinal tropism; industrial scalability	/	IBD, T1DM, SS	[41, 129]

#### 4.2.1 Systemic Lupus Erythematosus

SLE is a chronic systemic autoimmune disorder characterized by autoantibody production against self‐antigens, such as double‐stranded DNA and nuclear components. These autoantibodies form immune complexes that deposit in various tissues, leading to inflammation and organ damage [130, 131]. In addition to their diagnostic utility, EVs also exhibit significant therapeutic potential in SLE. For instance, MSC‐EVs have shown promise as therapeutic agents. Studies have indicated that BM‐MSC‐derived EVs can upregulate expression of anti‐inflammatory cytokines and alleviate SLE symptoms [76]. Li et al. demonstrated that EVs from human umbilical cord MSCs (hUCMSC‐EVs) restore the balance among Th1, Th17, and Treg cells suppress pathogenic double‐negative T cells (DNTs) and reduce plasma cell activity, thereby ameliorating renal injury in experimental LN models [55]. Moreover, MSCs may counteract SLE‐associated osteoporosis through epigenetic mechanisms, such as inhibition of the Notch signalling pathway, potentially reducing the risk of secondary osteoporosis [132]. MSC‐EVs have also been shown to promote autophagy in the inflammatory milieu of patients with SLE [133] and facilitate macrophage polarization by increasing phagocytic activity and recruiting Tregs, collectively contributing to attenuated disease progression [76].

#### 4.2.2 Rheumatoid Arthritis

RA is a chronic inflammatory disorder characterized by persistent synovitis, joint destruction, and systemic inflammation. Approximately half of all patients experience extra‐articular manifestations affecting the cardiac, pulmonary, ophthalmic, and hematologic systems [134]. Current investigations into EV‐based therapies for RA primarily leverage their innate or engineered immunomodulatory and targeting capacities. MSC‐EVs hold particular therapeutic promise [113]. Moreover, MSC‐EVs are more effective than blast cells and are particularly effective at inhibiting proinflammatory factors and the activation and differentiation of immune cells [135]. For instance, gingival MSC‐EVs (GMSC‐EVs) markedly reduce arthritis severity and bone erosion through suppression of the NF‐κB pathway, downregulation of IL‐17A expression, and upregulation of IL‐10 expression [76]. Similarly, EVs from M2‐polarised macrophages (M2‐EVs) promote the repolarization of proinflammatory M1 macrophages toward the anti‐inflammatory M2 phenotype, significantly ameliorating synovitis and joint damage in murine RA models without inducing systemic toxicity [136]. Adipose‐derived stem cell extracellular vesicles (ADSC‐EVs), which naturally carry diverse bioactive compounds, demonstrate pronounced anti‐inflammatory and tissue‐repair functions. Compared with cellular ADSC therapy, ADSC‐EVs have lower immunogenicity, better stability, and more convenient storage and handling requirements^1^ ^0^ ^3^. Advanced engineering strategies further increase EV applicability. Previous studies have shown that milk‐derived EVs coated with TNF‐α siRNA and attached to low‐temperature microneedles (cryoMNs) have good biocompatibility and can inhibit the proliferation of HFLS‐RA cells. They can effectively alleviate systemic RA conditions, changes in microcirculation indexes, pathological changes in synovial tissue, and the expression of related proteins in synovial tissue [137]. Interestingly, EVs from nonhuman sources, such as PELNs from ginseng, which transport plant microRNA to synovial macrophages to impede MAPK pathway phosphorylation, exhibit potential for use in RA therapy [124]. Advanced engineering strategies further increase EV applicability. Metabolic glycoengineering‐based surface modification prolongs EV circulation time and enables active CD44‐mediated active targeting to inflammatory sites, thereby promoting efficient accumulation in RA lesions [138]. Moreover, EV‐mimetic nanoparticles or EV membrane‐coated nanoparticles derived from RA‐relevant cells (e.g., synovial fibroblasts or macrophages) exploit natural homing motifs for targeted drug delivery to affected joints [139]. Furthermore, in vitro‐induced regulatory T‐cell‐derived extracellular vesicles (iTreg‐EVs) preferentially home to inflamed joints, where they rebalance Th17/Treg ratios and suppress effector T‐cell inflammation via Notch1 modulation, ultimately attenuating disease progression [116].

#### 4.2.3 Sjögren's Syndrome

Sjögren's syndrome is a chronic autoimmune disorder that primarily affects the exocrine glands, especially the salivary and lacrimal glands. It is pathologically defined by lymphocytic infiltration, which leads to xerostomia and keratoconjunctivitis sicca [140]. Research on EV‐based treatments for SS has concentrated largely on MSC‐EVs and engineered derivatives. MSCs and their EVs deliver bioactive molecules, such as TGF‐β1, PTX3, let‐7b‐5p, and miR‐21‐5p, that confer immunomodulatory effects and alleviate SS symptoms in preclinical models [111]. Notably, EVs from induced pluripotent stem cell‐derived MSCs (iPSC‐MSC‐EVs) suppress inflammatory episodes via immunomodulatory splenocytes, an effect increased by inhibition of miR‐125b [115]. To improve targeting and efficacy, researchers have developed let‐7f‐5p‐modified labial gland MSC‐EVs (LGMSC‐EVs), which restore the Th17/Treg balance by inhibiting the RORC/IL‐17A axis, leading to more effective symptom relief in patients with SS [141].

#### 4.2.4 Endocrine System

ADs of the endocrine system arise from immune dysregulation, which leads to targeted attack and functional impairment of the endocrine glands. Prominent examples include type 1 diabetes (T1D), Graves' disease, and autoimmune premature ovarian insufficiency (POI). EVs have emerged as promising therapeutic vehicles in this domain, primarily through immunomodulation and tissue repair. In T1D, EV‐based strategies focus on modulating immune responses and promoting β‐cell protection or regeneration. Human BMSC‐EVs increase Treg functionality, alleviate islet inflammation via small RNA transfer, and mitigate rejection in islet transplantation settings [142]. Additionally, murine serum‐derived EVs promote pancreatic β‐cell regeneration via delivery of miR‐106b‐5p and miR‐222‐3p, suggesting novel avenues for diabetes treatment [123]. In autoimmune POI, BMSC‐EVs enriched with miR‐21‐5p (miR‐21‐EVs) inhibit the apoptosis of ovarian granulosa cells by targeting MSX1 and modulating Notch signalling, thereby improving hormonal function and ameliorating POI pathology [112].

#### 4.2.5 Digestive System

ADs of the digestive system occur when the immune system attacks digestive organs such as the stomach, intestines, and liver, leading to structural damage and disruption of the local immune microenvironment. Growing evidence indicates that EVs from various sources can reduce inflammation, regulate immunological homeostasis, and aid in tissue healing. In liver‐related ADs, one innovative approach involves engineering M2 macrophage‐derived EVs to carry siRNA targeting receptor‐interacting protein kinase 3 (RIPK3). These modified EVs (M2 EVs/siRIPK3) significantly reduce liver injury in an autoimmune hepatitis (AIH) model, suppress RIPK3 expression, and decrease the levels of proinflammatory cytokines and chemokines in both liver tissue and serum [22]; The proteins and microRNAs enriched in milk‐derived EVs play an important roles in ulcerative colitis (UC) in mice. They regulate intestinal immune homeostasis by inhibiting the TLR4‐NF‐κB and NLRP3 signalling pathways, restoring the balance between Tregs and Th17 cells, and remodeling the intestinal microbiota. This alleviates colitis [143]. Furthermore, research has demonstrated that MEVs from both cow's milk and human milk can be absorbed by the intestine and can elicit comparable protective effects. They can both alleviate dextran sulfate sodium (DSS)‐induced colitis and reduce the histopathological score grade and degree of colon shortening. They also have therapeutic and anti‐inflammatory effects on colitis [144]. They can also promote the proliferation of intestinal epithelial cells, restore the integrity of the intestinal barrier and alleviate intestinal diseases and liver inflammation related to the gut‐liver axis, as well as metabolic diseases such as nonalcoholic steatohepatitis (NASH) [145].These findings underscore the promise of both natural and engineered EVs as therapeutic strategies for digestive ADs, primarily through immunomodulation, oxidative stress reduction, and increased tissue regeneration.

#### 4.2.6 Cutaneous System

ADs of the skin arise from misguided immune attacks on skin tissues, resulting in inflammation, damage, and clinical symptoms. As the body's largest organ and primary physical barrier, the skin plays a critical role in defense and its impairment compromises overall protection. These disorders are typically associated with hereditary and environmental factors, with common types being psoriasis, atopic dermatitis, and vitiligo. Accumulating evidence supports the therapeutic potential of EVs in skin ADs. In psoriasis, in which keratinocytes are attacked, MSC‐derived EVs reduce IL‐17 levels and terminal complement complex C5b‐9 deposition in mouse skin, mitigating neutrophil accumulation and IL‐17 release in the stratum corneum [146]. Additionally, small EVs from umbilical cord blood mononuclear cells (UCB‐MNC‐sEVs) reprogram macrophages to an anti‐inflammatory phenotype downregulate the expression of psoriasis markers (IL‐6, IL‐8, CXCL10, COX2, S100A7, and DEFB4) inhibit CD4^+^ and CD8^+^ T‐cell proliferation and promote Treg development via upregulation of FOXP3 expression, all of which collectively ameliorate disease severity [119]. In AD, which is characterized by Th2 cell overactivation, EVs from human adipose‐derived stem cells (ASC‐EVs) reduce serum IgE levels and eosinophil counts, ameliorate mast cell and macrophage (CD86^+^/CD206^+^) infiltration in skin lesions, and downregulate expression of proinflammatory genes (IL‐4, IL‐23, IL‐31, TNF‐α), supporting their potential as a new treatment avenue [114]. In vitiligo, which involves melanocyte destruction, EVs from 3D spheroid‐cultured human umbilical cord MSCs (3D‐EVs) promote Treg expansion and inhibit melanocyte apoptosis. Mechanistically, 3D‐EV‐enriched miR‐132‐3p and miR‐125b‐5p target Sirt1 and Bak1, respectively, attenuating oxidative stress and preserving melanocyte function [147].

#### 4.2.7 Nervous System

ADs of the nervous system are involved in immune‐mediated attacks on the central or peripheral nervous system, leading to neuroinflammation, neuronal degeneration, and functional impairments such as speech, visual, motor, and sensory deficits. Neuromyelitis optica spectrum disorders (NMOSDs), which are characterized by astrocyte injury, can be ameliorated by EV therapy. MSC‐derived EVs function by suppressing T‐cell activation, alleviating microglial and macrophage polarization, reducing oxidative stress, and playing a role in tissue regeneration and myelin membrane biogenesis, thereby effectively improving the secondary demyelination and inflammatory response caused by NMOSDs [148]. Another representative example is MS, characterized by demyelination. EVs have shown notable efficacy in reducing neuroinflammation and promoting remyelination. Various EV sources have demonstrated therapeutic effects in experimental autoimmune encephalomyelitis (EAE) models. Rhesus monkey MSC‐EVs inhibit the TLR2/IRAK1/NF‐κB pathway [121]; BMSC‐EVs reduce inflammatory cell infiltration into the CNS [128] and IFNγ‐stimulated human MSC‐EVs (IFNγ‐EVs) increase Treg populations [13]. Breast milk‐derived EVs can reduce the immune response in the central nervous system by decreasing the activation/phosphorylation of p38 MAPK and NF‐κB p50/p65 downstream of TLR4. This has significant positive implications for neuroinflammation, particularly in preterm infants [149]. Each of these EV types modulates microglial polarization, attenuates CNS inflammation, and mitigates demyelination, supporting their potential as innovative treatments for debilitating nervous system ADs.

#### 4.2.8 Ocular System

Thyroid eye disease (TED), autoimmune retinopathy (AIR), Sjögren's syndrome‐associated dry eye, and uveitis are examples of ocular ADs. Chronic inflammation, structural damage, and functional impairment of intraocular or periocular tissues are pathological features of these conditions. EVs offer considerable therapeutic advantages in this context, partly because of their ability to cross biological barriers, including the BOB. Our recent study demonstrated that circulating plasma‐derived EVs involved in systemic regulation respond to RGC degeneration in patients with glaucoma [120]. In uveitis, MSC‐EVs suppress Th1 and Th17 cell differentiation in mixed lymphocyte reactions and inhibit the activation of antigen‐presenting cells (APCs) and T lymphocytes [150]. These immunomodulatory mechanisms collectively contribute to delayed disease progression and reduced intraocular inflammation. With respect to Sjögren's syndrome‐related dry eye, studies by both the Aluri and Abughanam groups have demonstrated that the systemic administration of MSC‐EVs in NOD mouse models leads to the sustained suppression of ocular inflammation and the enhancement of tissue repair mechanisms [151].

#### 4.2.9 Other Autoimmune Diseases

EVs also exhibit considerable therapeutic potential in organ‐specific ADs affecting the urinary system. In LN, BMSC‐EVs ameliorate renal inflammation and injury in MRL/lpr mice by upregulating the expression of anti‐inflammatory mediators such as Arg‐1 and CCL20, thereby promoting macrophage polarization toward an anti‐inflammatory phenotype and conferring renal protection [76]. In the field of diabetic nephropathy, human urine‐derived stem cell‐derived EVs (USC‐EVs) have been demonstrated to prevent kidney complications associated with type 1 diabetes. Treatment with USC‐EVs reduces urine output and microalbuminuria, suppresses podocyte and renal tubular epithelial apoptosis via caspase‐3 downregulation, and promotes the proliferation of glomerular endothelial cells [98]. In response to acute kidney injury (AKI), mEVs reduce oxidative stress and inflammation through the delivery of siRNAs that target protein tyrosine phosphatase 1B, which restored the integrity of the tight junctions in kidney tissue, highlighting the therapeutic potential of mEVs in regulating key pathological processes in AKI [152].

### 4.3 Therapeutic Applications of Plant‐Derived Extracellular Vesicle‐Like Nanoparticles

Plant‐derived exosome‐like nanoparticles (PELNs) share structural similarities with mammalian EVs but offer distinct advantages: lower immunogenicity, reduced production costs, natural sustainability, and absence of human pathogens [153]. These properties make PELNs promising carriers for therapeutic agents or poorly soluble natural compounds. However, their direct application in ADs remains limited. This section focuses on their role in digestive system ADs and outlines progress in related diseases to inform future research.

In IBD, PELNs exhibit considerable therapeutic potential through multiple mechanisms. Their anti‐inflammatory and antioxidant effects are well‐documented. For instance, PELNs from Portulaca oleracea L. accumulate in inflamed colon tissue, suppressing pro‐inflammatory cytokines and elevating IL‐10 [125]. Turmeric‐derived PELNs inactivate the NF‐κB pathway, downregulating pro‐inflammatory factors and upregulating antioxidant genes like HO‐1 [128]. Ginseng‐derived PELNs scavenge reactive oxygen species in immune cells and intestinal epithelial cells, exerting anti‐inflammatory effects via modulation of pathways such as TLR4/MAPK [53]. Additionally, grapefruit‐derived PELNs are selectively taken up by intestinal macrophages, elevate HO‐1 expression, and inhibit IL‐1β and TNF‐α production [30], thereby contributing to the maintenance of intestinal macrophage homeostasis. Broccoli PELNs modulate immune responses by activating AMP‐activated protein kinase (AMPK) and suppressing excessive dendritic cell (DC) activation [126]. Beyond immunomodulation, PELNs also play important roles in intestinal barrier repair and gut microbiota remodeling [154]. For instance, tea leaf‐derived PELNs restore the damaged colonic barrier through galactose group‐mediated specific macrophage endocytosis and enhance gut microbial diversity and overall abundance [155]. Honeysuckle PELNs increase short‐chain fatty acids, regulate bile acid metabolism, and alleviate colitis [156]. Similarly, Garlic PELNs inhibit TLR4/MyD88/NF‐κB and modulate gut microbiota [157]. Grape PELNs leverage their unique lipid composition to stimulate the proliferation of Lgr5+ intestinal stem cells, promoting intestinal tissue repair and regeneration [127].

Currently, PELN applications in other ADs are exploratory but promising. In RA, ginseng PELNs deliver plant miRNAs to synovial macrophages, suppressing MAPK pathway phosphorylation [124]. Folate‐modified ginger PELNs target M1 macrophages, promoting M2 polarization via PI3K‐AKT [158]. In cutaneous immunity, purslane PELNs modulate the M1/M2 polarization balance via NF‐κB/STING pathways, improving atopic dermatitis [159]. Perilla leaf PELNs in a hydrogel formulation deliver miRNA to inhibit IL‐17 signaling, alleviating psoriasis [160].

Notably, PELNs show encouraging results in disease models with pathogenesis similar to ADs. For example, ginger‐derived PELNs alleviate pulmonary inflammation by delivering miR‐396a‐5p [161] and inhibit the activation of the NLRP3 inflammasome, a key regulator of innate immune responses [162]. They also activate the Nrf2 pathway, enhancing hepatic detoxification [163]. Strawberry PELNs protect human mesenchymal stromal cells from oxidative damage [164], while blueberry PELNs mitigate non‐alcoholic fatty liver disease by attenuating mitochondrial oxidative stress [165]. Garlic PELNs carry miRNA that regulates macrophage metabolism and alleviates obesity‐related inflammation [166]. Balloon flower PELNs downregulate inflammatory factors and modulate macrophage polarization, improving acute lung injury [167]. Cabbage‐derived PELNs inhibit immune cell inflammation and apoptosis in human keratinocytes and fibroblasts, suggesting their potential as novel biomaterials for therapeutic applications [168].

## 5 Current Challenges in Clinical Application

To advance the clinical translation of EVs as therapeutic and drug delivery platforms, it is essential to systematically identify and address the core challenges associated with scalable production, quality control, and in vivo performance. These challenges primarily include heterogeneity in donor sources and production processes, lack of standardization in isolation and purification techniques, insufficient in vivo targeting and barrier penetration capabilities, limitations in pharmacokinetics and stability, and an underdeveloped framework for systematic safety and immunogenicity assessment. In this section, these critical bottlenecks are analyzed, and potential strategies and future directions to overcome them are discussed.

### 5.1 Donor Source Heterogeneity

The biological properties and functions of EVs are profoundly influenced by their cellular origin, making donor heterogeneity a fundamental challenge for achieving product consistency. With respect to animal‐derived EVs, factors such as the donor's age, sex, genetic background, and physiological status significantly affect the bioactive components (e.g., proteins, RNA, and lipids), leading to functional variations between batches. Recent studies have indicated that even small EVs secreted by different cell types derived from the same stem cell line (e.g., human pluripotent stem cells) exhibit distinct surface protein marker profiles. These findings underscore the importance of defining and tracking the “cellular identity” of EVs during production to ensure functional consistency [169].

PELNs are notably influenced by species, tissue source (e.g., fruit, leaf, and root), and growth conditions (e.g., light, soil composition, and seasonal variations), resulting in unstable enrichment levels of secondary metabolites such as curcuminoids and flavonoids within the vesicles [18]. To mitigate the variability introduced by donor heterogeneity, it is necessary to establish stringent screening and characterization criteria for donor cells or plant materials. For animal cells, the use of well‐characterized, standardized cell lines with clear genetic backgrounds is recommended. For PELNs, standardized agricultural cultivation and management practices are required to stabilize the quality of the raw material quality. Concurrently, batch testing of key active components is imperative.

### 5.2 Standardization of Isolation and Purification Techniques

The absence of a unified, efficient, and scalable gold standard method for EV isolation is a major technological bottleneck hindering clinical translation. Techniques used by different laboratories or production platforms, such as ultracentrifugation, size‐exclusion chromatography [27], sucrose density gradient centrifugation [170], and immunaffinity capture [12], vary in principle and yield final products with significant differences in purity, subpopulation ratios, surface protein integrity, and coisolated impurities (e.g., protein aggregates and lipoproteins). Research has shown that EVs isolated from the same cell source can exhibit severalfold differences in marker abundance when different methods are used [171]. This heterogeneity, directly introduced by the purification workflow, makes it difficult to directly compare or reproduce data and therapeutic efficacy across different studies or production batches.

While current guidelines from the International Society for Extracellular Vesicles (ISEV) recommend a combination of nanoparticle tracking analysis (NTA), transmission electron microscopy (TEM), and western blotting for characterization, these methods have limitations, and a globally standardized quality control framework has yet to be established. The lack of comprehensive preclinical data and regulatory approval standards for plant EVs, in particular, severely impedes their clinical translation. Beyond traditional standardization systems, there is a pressing need to establish novel quality control standards specifically tailored to EV markers.

### 5.3 In Vivo Targeting and Barrier Penetration

Effective therapeutic outcomes require EVs to precisely home to lesion sites and surmount physiological barriers, areas in which naturally occurring EVs exhibit inherent limitations. With respect to barrier penetration, although membrane‐coated nanostructures confer inherent penetrative potential to EVs [172, 173], particularly those of animal origin [174], plant‐derived EVs often exhibit limited permeability because of the absence of human‐specific receptor recognition systems and heterogeneity in particle size, hindering their ability to traverse physiological barriers [170] such as the BBB [175] and BOB [176]. Enhancement strategies include employing focused ultrasound (FUS) [177] combined with microbubbles to transiently open the BBB and facilitate the cerebral delivery of EVs, modifying EV membranes with cell‐penetrating peptides (CPPs) [178] to mediate efficient intracellular transport, and applying electric fields or acoustic waves to perturb cell membrane permeability, thereby increasing cellular uptake efficiency.

### 5.4 In Vivo Stability and Pharmacokinetics

In terms of in vivo stability post administration, EVs often display insufficient stability, a short half‐life, and rapid clearance because of hepatic and splenic sequestration. Potential solutions include developing sustained‐release delivery platforms through strategies such as loading EVs into engineered multifunctional hydrogel matrices [179], integrating them with porous microspheres [179] or 3D‐printed microneedle arrays [180] for customized scaffold fabrication [181], or encapsulating EVs within bioactive nanomaterial‐functionalized [182] hyaluronic acid hydrogel microspheres [183]. These integrated approaches enable controlled EV release while concurrently increasing local immunomodulation and inflammatory microenvironment remodeling. Recent studies have demonstrated the use of biodegradable polymer microcapsules loaded with EVs for the treatment of vitreoretinal disease, providing new avenues for sustained intraocular delivery [184].

Additionally, some natural EVs lack cell selectivity and are susceptible to nonspecific uptake by nontargeted cells [185]; natural ligands on the surface of PELNs have difficulty effectively recognizing mammalian receptors. Traditional ligand fusion strategies are often ineffective because of in vivo degradation or conformational changes that occur. Novel targeting strategies include the precise assembly of specific nanobodies on the surface of EVs using a glycosylphosphatidylinositol (GPI) anchoring system, which significantly increases their targeting ability, and the modification of EVs [186] with ligand‐coupled polyethyleneglycol (PEG)‐derived phospholipids, which simultaneously increases their cell‐specific recognition and prolongs their in vivo cycling time, resulting in an increase in the accumulation of target tissues and the efficiency of drug delivery [187].

### 5.5 Safety and Immunogenicity Assessment

As they are exogenous biological products, the safety profile of EVs for clinical application is paramount; however, the assessment framework remains underdeveloped. Challenges stem from their compositional complexity, potential immunogenicity, and unknown long‐term biological safety. PELNs, while circumventing the risk of animal pathogens, carry a rich array of plant‐derived bioactive molecules (e.g., specific proteins and secondary metabolites). Their immunorecognition, metabolic fate, and potential for cross‐reactivity in humans constitute largely unexplored territory and may trigger unintended immune or metabolic responses [170]. In contrast, the immunogenicity challenges associated with animal‐derived EVs are more complex and multifactorial. Beyond potentially carrying donor cell‐associated antigens, their immunogenicity depends heavily on the source (autologous, allogeneic, or xenogeneic), size, surface molecular composition (e.g., a high abundance of MHC‐I molecules may trigger adaptive immune responses), internal cargo, preparation and storage methods (e.g., harsh ultracentrifugation may cause vesicle damage and aggregation), and dosing regimen (e.g., repeated administration may induce an accelerated blood clearance phenomenon) [188].

Addressing these challenges requires the establishment of a systematic safety evaluation framework. This includes comprehensive immunogenicity profiling of EVs (e.g., cytokine release, complement activation and lymphocyte proliferation assays) and identification of key active and potential risk components via proteomics and metabolomics [189]. Preliminary evidence of the safety of animal‐derived EVs is emerging from early‐phase clinical trials (Table 5). For PELNs, special attention must be given to assessing the human safety of plant‐specific components. Long‐term safety studies should focus on their in vivo distribution, clearance mechanisms, and potential impact on physiological homeostasis [190].

## 6 Future Prospects

### 6.1 Multiomics Study

**FIGURE 5:**
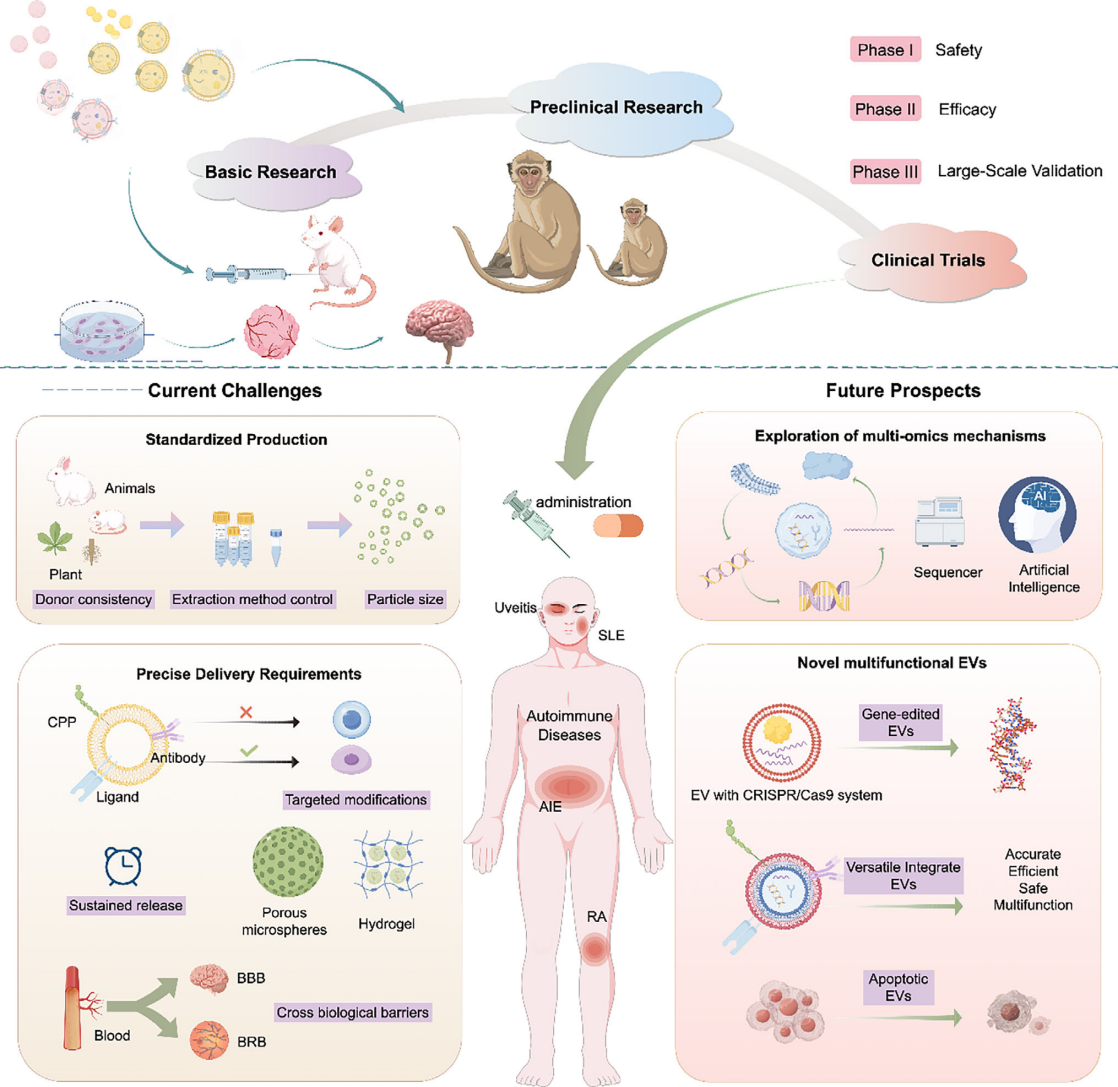
Challenges and Prospects in the Clinical Translation of Extracellular Vesicles. The uppermost layer illustrates a continuum of translation from fundamental research through animal model validation and preclinical studies to clinical trials, ultimately resulting in application to AD patients and the formation of a patient‐centered closed loop. The bottom panel summarizes the present challenges in EV standardization and targeted delivery, alongside future prospects emphasizing multiomics studies and multifunctional engineered EVs. **Abbreviations**: EVs, extracellular vesicles; CPP: cell‐penetrating peptide; BBB: blood–brain barrier; BRB: blood–retinal barrier; SLE: systemic lupus erythematosus; IBD: inflammatory bowel disease. Created in FigureDraw. https://www.figdraw.com/#/.

In‐depth investigations into the molecular mechanisms of EVs are essential for expanding their therapeutic potential. Characterizing dynamic transcriptomic changes within immune cell subsets, such as T cells, macrophages, and dendritic cells, following EV treatment can reveal critically regulated signalling pathways and allow real‐time monitoring of EV‐mediated immune responses [49]. For instance, Netrin‐1‐modified endothelial cell‐derived EVs promote neural repair, and multiomics analyses have revealed their roles in activating core processes related to neural niche formation, including adhesion plaque assembly, axon guidance, and PI3K–AKT and mTOR signalling pathways [191]. These findings underscore the promise of engineered EVs in mitigating neurological damage associated with ADs. Moreover, disease‐specific multiomics profiling, such as in models of sepsis, offers a foundational dataset for developing tailored EV‐based therapeutic platforms [192]. Molecular profiling of EVs from patients with ADs may therefore yield new insights into EV‐mediated anti‐inflammatory mechanisms. Concurrently, longitudinal monitoring of circulating EV‐derived RNAs via liquid biopsy enables dynamic assessment and prediction of therapeutic efficacy [193]. The elucidation of these mechanisms through multiomics approaches directly supports strategies for regulating pathological EV release, which is an emerging therapeutic strategy for disease modulation (Figure 5). Accumulating evidence indicates that tumor‐derived EVs and particles (EVPs) play a key role in promoting hepatocellular carcinoma progression. Notably, ablation of Kupffer cells or blockade of TNF signalling has been shown to significantly attenuate their protumorigenic effects [194]. Given the growing interest in EVs as mediators of cancer immunotherapy, these findings may also extend to the treatment of ADs. For instance, STING agonists have been shown to induce potent antitumor immune activation [195]. Conversely, immune suppression is often desirable in autoimmune and inflammatory contexts; thus, STING inhibitors such as H‐151 may offer novel therapeutic opportunities for ADs. Integrating multiomics data thus enables the identification of key molecular targets for inhibiting pathogenic EV release and allows precise regulation of EV‐mediated processes across diseases.

### 6.2 Multifunctional Engineered Extracellular Vesicles

The controlled coencapsulation of gene‐editing tools (such as the CRISPR/Cas9 system [196]) and auxiliary therapeutic molecules into EVs enables multitargeted synergistic therapy. Engineered EV delivery platforms constructed using methods such as protein heterodimerization [197], ultrasound‐assisted loading, and other advanced techniques have demonstrated efficacy in models of genetic disorders, inflammatory diseases, neurodegenerative conditions, and tumors [44]. Nevertheless, EV‐based gene editing continues to face major challenges, including low loading efficiency, limited production scalability, and the need for cross‐species safety validation. Future research should aim to overcome these engineering barriers, foster interdisciplinary collaboration, and accelerate the translation from preclinical models to clinical use to realize precise, efficient, and safe therapeutic applications. Promising strategies include multicomponent coloading techniques, synergistic delivery systems, and innovative hybrid engineered platforms [198], such as biononbio membrane nanotechnology fusion [199] and dual synergistic nanoregulators [200].

## 7 Conclusion

The intricate pathogenesis of ADs, coupled with physiological tissue barriers and the constraints of conventional therapeutics, has led to significant treatment challenges, thereby accelerating investigations into both disease mechanisms and targeted drug development. EVs, as innate nanoscale mediators of intercellular communication and cargo delivery, have demonstrated considerable promise in the diagnosis and treatment of ADs, particularly as diagnostic biomarkers, immunomodulatory agents, and drug delivery vehicles. This review systematically outlined the mechanisms and therapeutic applications of EVs in ADs, highlighting their clinical translational value as well as persistent obstacles. Owing to their intrinsic targeting capacity and protective encapsulation of biomolecules, EVs play dual roles as both “immune homeostasis modulators” and “targeted delivery systems.” They integrate therapeutic delivery, immunomodulation, and tissue repair functions into a single platform, enabling them to overcome biological barriers and facilitate targeted intervention. EV research is promoting a shift in AD treatment paradigms from broad‐spectrum immunosuppression to precision immune reprogramming. However, clinical implementation remains contingent upon addressing two major challenges: standardized manufacturing protocols and increased targeting specificity and delivery efficiency. In the future, advanced engineering approaches are expected to yield novel “smart” EVs capable of serving as customizable therapeutics. Such progress could transform the management of ADs by enabling the early detection of biomarkers and the development of personalized, EV‐based treatments, thereby paving the way for precision medicine and substantially improving clinical outcomes.

**TABLE 5:** Summary of EVs‐based Clinical Trials in Autoimmune Diseases.

Company/Medical Center/University (Location)	EVs	Disease/Condition	Clinical Trial Number	Clinical Trial Phase/Status (Start/Completion Date)	Summary
Xuanwu Hospital, Beijing; ECHO Biotech (No specific location)	Human umbilical cord mesenchymal stem cell‐derived EVs (hUC‐MSC‐Exos)	Autoimmune encephalitis	NCT07131683	Phase I/IIa, NOT_YET_RECRUITING (2025‐11‐01)	To investigate the safety and preliminary efficacy of intranasal administration of hUC‐MSC‐Exo in patients with autoimmune encephalitis
General Committee of Teaching Hospitals and Institutes, Egypt (Sahel, Shubrā, Cairo Governorate)	Umbilical cord blood‐derived MSC microvesicles	Type 1 diabetes mellitus (T1DM)	NCT02138331	Phase II/III, UNKNOWN (2014‐04)	To evaluate the effect of intravenous infusion of cell‐free MSC microvesicles on inflammatory state, β‐cell mass and glycemic control in T1DM patients [201]
Biocells Medical (Warsaw, Poland)	Stem cell‐derived biologics (including MSCs and EVs)	Autoinflammatory and postinfectious neuroinflammatory syndromes	NCT07145502	ACTIVE_NOT_RECRUITING, Observational Registry Study (2022‐09‐19)	To track improvements in neurological function, inflammatory biomarkers and patient‐reported quality of life in patients receiving the biologics
Biocells Medical (Warsaw, Poland)	Allogeneic mesenchymal stem cell‐derived EVs	Progressive multiple sclerosis (MS)	NCT07146087	EARLY_PHASE1, SUSPENDED (2020‐07‐01)	To assess the effect of intravenous EVs infusion on disability progression and neurological function, as well as the safety and tolerability of repeated infusions
Iran University of Medical Sciences (Moheb Kowsar Hospital, Tehran, Iran)	Limbal stem cell‐derived EVs (LSC‐Exos)	Dry eye	NCT06543667	Phase I, RECRUITING (2024‐08‐01)	To determine the safety and efficacy of LSC‐Exo eye drops in alleviating dry eye symptoms [202]
RenJi Hospital, Shanghai Jiaotong University School of Medicine (Shanghai, China)	Urinary EVs	Lupus nephritis (LN)	NCT04894695	UNKNOWN (2020‐08‐02)	To perform transcriptome and/or metabonomics sequencing of urinary EVs and screen for differential molecules specific to LN patients [203]
First Affiliated Hospital of Jinan University (Guangzhou, Guangdong, China)	Serum exosomal miRNA	Ocular myasthenia gravis (OMG)	NCT05888558	UNKNOWN (2023‐07‐04)	To screen specifically expressed miRNAs as biomarkers for OMG diagnosis
Liga Panamericana de Asociaciones de Reumatologia (PANLAR); Janssen Research & Development, LLC (Rosario, Argentina)	Serum and urinary biomarkers (including EVs‐related subgroup study)	Systemic lupus erythematosus (SLE), lupus nephritis (LN)	NCT04534647	UNKNOWN (2019‐06‐01)	To evaluate the correlation between serum/urinary biomarkers and socio‐demographic, clinical and serological manifestations of the disease; transcriptome studies will be conducted in subgroups
Aegle Therapeutics (No specific location)	Bone marrow mSC‐derived EVs (BM‐MSC‐Exos, product name: AGLE‐102)	Dystrophic epidermolysis bullosa (EB)	NCT04173650	Phase I/II (Phase II mentioned in some sources), start date not specified	A Phase I/II open‐label single‐group trial to test the safety and efficacy of BM‐MSC‐Exos (AGLE‐102) on lesions of EB patients; 10 patients aged 6 years or older with a single wound will be given 6 doses over 3 months [204]
Direct Biologics, LLC (no specific location)	ExoFlo (Ex vivo culture‐expanded adult allogeneic bone marrow mesenchymal stem cell‐derived extracellular vesicle isolate product)	Medically refractory ulcerative colitis	NCT05176366	Phase 1, COMPLETED (started 2022‐12‐19, completed 2024‐12‐01)	A Phase 1 multi‐site open‐label trial to evaluate the feasibility of intravenous ExoFlo in patients with medically refractory ulcerative colitis; 4 patients enrolled, subject participation period 58 weeks, study duration 24 months
University of Louisville (Louisville, USA)	Ginger plant EVs (alone or combined with curcumin)	Inflammatory bowel disease (Including crohn disease, ulcerative colitis)	NCT04879810	Phase not applicable, COMPLETED (started 2018‐03‐01, completed 2022‐08‐02)	An exploratory randomized study testing ginger plant EVs alone, curcumin alone, and their combination in patients with inflammatory bowel disease; to evaluate safety, tolerability, and effects on symptoms, disease scores, and inflammatory biomarkers; planned to enroll 90 chronic IBD patients (3 arms of 30), 4 patients actually enrolled (eligible clinic/referred patients) [96]
Taipei Veterans General Hospital (Taipei, Taiwan)	Exosomal miRNA (source not specified)	Dry eye syndrome, sjögren's syndrome	NCT06475027	NOT_YET_RECRUITING (2024‐07‐01)	To analyze exosomal miRNA and transcriptome profiles after acupuncture and Chinese herbal medicine treatment, and explore the mechanism of therapeutic action [202]
Taipei Veterans General Hospital (Taipei, Taiwan)	EVs (source not specified)	Sjögren's syndrome (SJS), dry eye syndrome (DES)	NCT06771427	RECRUITING (2025‐01‐16)	To analyze differential proteins in exosomes, clarify disease pathological mechanisms, and explore potential diagnostic and therapeutic methods [205]

## Funding

This research was supported by the National Natural Science Foundation of China under Grant No. 82271114, and the Zhejiang Provincial Medical and Health Science and Technology Plan under Grant No. WKJ‐ZJ‐2511.

## Financial Disclosures

The authors have no proprietary interest in any materials or methods described within this article.

## Conflicts of Interest

The authors declare no conflict of interest.
